# Generation of the tumor-suppressive secretome from tumor cells

**DOI:** 10.7150/thno.61006

**Published:** 2021-07-25

**Authors:** Shengzhi Liu, Xun Sun, Kexin Li, Rongrong Zha, Yan Feng, Tomohiko Sano, Chuanpeng Dong, Yunlong Liu, Uma K. Aryal, Akihiro Sudo, Bai-Yan Li, Hiroki Yokota

**Affiliations:** 1 Department of Biomedical Engineering, Indiana University Purdue University Indianapolis, Indianapolis, IN 46202, USA; 2 School of Pharmaceutical Sciences, Capital Medical University, Beijing 100069, China; 3 Department of Pharmacology, School of Pharmacy, Harbin Medical University, Harbin 150081, China; 4 Department of Orthopedic Surgery, Mie University, Mie 514, Japan; 5 Center for Computational Biology and Bioinformatics, Department of Medical and Molecular Genetics, Indiana University School of Medicine, Indianapolis, IN 46202, USA; 6 Department of Comparative Pathobiology, Purdue University, West Lafayette, IN 47907, USA; 7 Indiana Center for Musculoskeletal Health, Indiana University School of Medicine, Indianapolis, IN 46202, USA; 8 Simon Cancer Center, Indiana University School of Medicine, Indianapolis, IN 46202, USA

**Keywords:** breast cancer, β-catenin, Wnt signaling, enolase 1, ubiquitin C

## Abstract

**Rationale**: The progression of cancer cells depends on the soil and building an inhibitory soil might be a therapeutic option. We previously created tumor-suppressive secretomes by activating Wnt signaling in MSCs. Here, we examined whether the anti-tumor secretomes can be produced from tumor cells.

**Methods:** Wnt signaling was activated in tumor cells by overexpressing β-catenin or administering BML284, a Wnt activator. Their conditioned medium (CM) was applied to cancer cells or tissues, and the effects of CM were evaluated. Tumor growth in the mammary fat pad and tibia in C57BL/6 female mice was also evaluated through μCT imaging and histology. Whole-genome proteomics analysis was conducted to determine and characterize novel tumor-suppressing proteins, which were enriched in CM.

**Results:** The overexpression of β-catenin or the administration of BML284 generated tumor-suppressive secretomes from breast, prostate and pancreatic cancer cells. In the mouse model, β-catenin-overexpressing CM reduced tumor growth and tumor-driven bone destruction. This inhibition was also observed with BML284-treated CM. Besides p53 and Trail, proteomics analysis revealed that CM was enriched with enolase 1 (Eno1) and ubiquitin C (Ubc) that presented notable tumor-suppressing actions. Importantly, Eno1 immunoprecipitated CD44, a cell-surface adhesion receptor, and its silencing suppressed Eno1-driven tumor inhibition. A pan-cancer survival analysis revealed that the downregulation of MMP9, Runx2 and Snail by CM had a significant impact on survival outcomes (*p* < 0.00001). CM presented a selective inhibition of tumor cells compared to non-tumor cells, and it downregulated PD-L1, an immune escape modulator.

**Conclusions:** The tumor-suppressive secretome can be generated from tumor cells, in which β-catenin presented two opposing roles, as an intracellular tumor promoter in tumor cells and a generator of extracellular tumor suppressor in CM. Eno1 was enriched in CM and its interaction with CD44 was involved in Eno1's anti-tumor action. Besides presenting a potential option for treating primary cancers and metastases, the result indicates that aggressive tumors may inhibit the growth of less aggressive tumors via tumor-suppressive secretomes.

## Introduction

A chemotherapeutic drug is generally an inhibitor of a tumor-stimulating pathway such as Wnt signaling, PI3K signaling, and EMT and the use of any activator of Wnt or PI3K is hardly considered a therapeutic option. To explore the unconventional territory, however, we evaluated the role of those activators in the tumor microenvironment. In tumor-osteocyte interactions, in which bone formation is promoted by Wnt signaling, we reported that the overexpression of β-catenin and Lrp5, a Wnt co-receptor, granted osteocytes anti-tumor capabilities 1. This anti-tumor capability was also observed in MSCs that overexpressed varying protumorigenic genes such as β-catenin, Lrp5, snail and Akt. We named those osteocytes and MSCs induced tumor-suppressing cells (iTSCs). Cell culture and preclinical studies have revealed that iTSCs generate anti-tumor secretomes and their complete and partial conditioned medium (CM) inhibits the progression of tumor cells *in vitro*, *ex vivo* and *in vivo* 1-3. The question herein was whether the anti-tumor secretomes can be generated not only from non-tumor cells but also from tumor cells.

In this study, we focused on canonical Wnt signaling that plays a pivotal role in cellular signaling and homeostasis in many tissues, including loading-driven bone formation 4, 5. The derailed activation of Wnt signaling is reported to promote the progression of tumors and many studies have been directed to inhibit Wnt activation for cancer treatments 6, 7. We have shown that the overexpression of Lrp5, a Wnt co-receptor, stimulated the proliferation and migration of breast cancer cells 1. To our surprise, however, we have also shown that the conditioned medium, collected from Lrp5-overexpressing osteocytes and MSCs, can suppress tumor growth 2. Collectively, Wnt signaling presented a conflicting pair of actions as an intracellular tumor promoter in tumor cells and a generator of tumor-suppressing proteins in osteocytes and MSCs. The question herein was whether the activation of Wnt signaling in tumor cells generates tumor-promotive or tumor-suppressive secretome. We hypothesized that the overexpression of a Wnt activator, such as β-catenin, generates an aggressive tumor clone that synthesizes and secretes tumor-suppressing proteins in CM and eliminates neighboring less aggressive tumor clones.

To conduct a proof-of-principle test of the above hypothesis, β-catenin was overexpressed in mammary tumor cells, and their CM was applied to tumor cells, cancer-tissue fragments, and tumor-inoculated mice. A synthetic activator of Wnt signaling, BML284 8, 9, was also employed. The result showed that β-catenin and BML-284 were double-edged swords that acted oppositely in tumor cells and their microenvironment. As a pair of negative control genes, we selected the Wnt1, a ligand, and Fzd7, a co-receptor in Wnt signaling, and overexpressed them in tumor cells. Whole-genome proteomics analysis revealed that a pair of unexpected proteins, enolase 1 (Eno1) and Ubc, were enriched in CM and they acted as remarkable tumor suppressors in the extracellular domain. Tumor cell-derived CM, as well as atypical tumor suppressors such as extracellular Eno1 and Ubc, preferentially inhibited the viability of tumor cells over non-tumor cells, and they downregulated programmed death-ligand1 (PD-L1), one of the targets of immunotherapy 10.

To evaluate the potential impact of CM administration, we conducted a pan-cancer survival analysis focusing on the regulation of three genes such as MMP9, Runx2, or Snail. We also performed an immunoprecipitation assay to elucidate the mechanism of Eno1's anti-tumor action. Eno1 immunoprecipitated CD44, a transmembrane adhesion receptor, whose expression has been shown to correlate with both favorable and unfavorable clinical outcomes 11. We examined CD44 since it is known as a major Wnt target gene and its expression is linked to Wnt activity 12. Collectively, the present study suggests a possibility of employing cancer cells and protumorigenic factors to treat cancer. Besides popularly administered chemotherapy that mostly inhibits specific oncogenic signaling, this study indicates a novel option with iTS cells and their complete or partial CM that are generated by activating a protumorigenic pathway such as Wnt signaling. The result herein also sheds light on a century-old paradoxical observation, in which influential tumors may inhibit the progression of less aggressive tumors and their surgical removal could adversely affect the survival of patients.

## Materials and Methods

**Cell culture and agents.** MLO-A5 osteocyte-like cells (obtained from Dr. L. Bonewald, Indiana University, IN, USA) and RAW 264.7 pre-osteoclast cells (ATCC, Manassas, VA, USA) were cultured in α-MEM (12571048, Thermo Fisher Scientific, Waltham, MA, USA). EO771 mouse mammary tumor cells (CH3 BioSystems, Amherst, NY, USA), 4T1.2 mouse mammary tumor cells (obtained from Dr. R. Anderson at Peter MacCallum Cancer Institute, Melbourne, Australia), PANC-1 human pancreatic cancer cells (ATCC), and MCF-7 human estrogen receptor (ER)-positive breast cancer cells (ATCC) were cultured in DMEM (MT10013CV, Thermo Fisher Scientific). TRAMP-C2ras prostate tumor cells (ATCC) were cultured in DMEM/F-12 13, and MDA-MB-231 human estrogen receptor (ER)-negative breast cancer cells and PC-3 human prostate cancer cells (ATCC) were cultured in RPMI-1640 (15040CV, Corning, Glendale, Arizona, USA) 14, 15. As non-tumor control cells, two epithelium lines (KTB36 and KTB6, obtained from Dr. H. Nakshatri, Indiana University) with the breast epithelial origin were cultured in F12-DMEM (low glucose) in a 3:1 ratio. For the tumor cells, the culture media were supplemented with 10% fetal bovine serum (FBS) (S11550H, Bio-Techne, Minneapolis, MN, USA) and antibiotics (50 units/mL penicillin, and 50 μg/mL streptomycin; 15140122, Life Technologies, Grand Island, NY, USA). For MLO-A5 cells, the media contained 5% FBS, 5% fetal calf serum, and antibiotics. Cells were maintained at 37°C and 5% CO_2_.

Eno1 (500 ng/mL, MBS2009113), and Ubc (500 ng/mL, MBS2029484, MyBioSource, San Diego, California, USA) recombinant proteins were given to EO771 cells, and cells were incubated for 24 h. A pharmacological inhibitor of Eno1 (ENOblock - AP-III-a4, MBS385406, MyBioSource) and an inhibitor of E3 ubiquitin ligase (Pomalidomide, MBS8005710, MyBioSource) were applied to the cells for 24 h 16, 17.

**EdU assay.** Cellular proliferation was examined using a fluorescence-based cell proliferation kit (C10337, Click-iT™ EdU Alexa Fluor™ 488 Imaging Kit; Thermo Fisher Scientific). Approximately 2,000 cells were seeded in 96-well plates (3585, Corning) on day 1, CM and drugs were given on day 2, and cells were labeled with 10 μM EdU on day 4 for 4 h. After labeling, cells were fixed in a 3.7% (w/v) formaldehyde solution for 15 min at room temperature. They were washed with a PBS buffer (3% BSA, 0.5% Triton® X-100) and incubated with a freshly prepared Click-iT® reaction cocktail in dark for 30 min. After rinsing with a PBS buffer, eight images from four wells in each group were taken with a fluorescence microscope (magnification, 100×, Olympus, Tokyo, Japan). The number of fluorescently labeled cells, as well as the total number of cells, were counted using Image J (National Institutes of Health, Bethesda, MD, USA) and the ratio of the fluorescently labeled cells to the total cells was determined 18.

**MTT assay.** Approximately 2,000 cells were seeded in 96-well plates (3585, Corning) on day 1, CM and drugs were given on day 2, and cells were dyed with 0.5 mg/mL thiazolyl blue tetrazolium bromide (M5655, Sigma, St. Louis, MO, USA) on day 4 for 4 h. Optical density for assessing metabolic activities was determined at 570 nm using a multi-well spectrophotometer. The relative cell viability was determined as an absorbance ratio of each sample to a control.

**Invasion assay.** The invasion capacity of cancer cells was determined using a 24-well plate and transwell chambers (352097, Thermo Fisher Scientific) with 8-μm pore size. Transwell chambers were coated with 150 μL Matrigel (100 μg/mL) that was polymerized and dried overnight. Three-hundred μL of the serum-free medium was added to each chamber and after 1 h, the chamber was washed three times with the serum-free medium. Approximately 5×10^4^ cells in 200 μL serum-free DMEM were then placed on the upper chamber and 800 μL iTS CM was added to the lower chamber. After 48 h, the cells on the upper surface of the membrane were removed and the membrane was treated with ~400 μL of 75% ethanol in a fresh 24-well plate for 40 min. The cells, which invaded the lower side of the membrane, were stained with Crystal Violet (diluted 1:25 in water) for 30 min. At least five randomly chosen images were taken with an inverted optical microscope (100× magnification, Nikon, Tokyo, Japan), and the average number of stained cells, which represented the invasion capacity, was determined.

**Two-dimensional motility assay.** A wound-healing scratch motility assay was performed to assess 2-dimensional cell motility. Approximately 3×10^5^ cells were seeded in 12-well plates, and after the cell attachment, a scratch was made on the cell layer with a plastic pipette tip. Cell medium was exchanged and floating cells were removed. Images of the cell-free areas were captured at 0 h and 24/48 h after scratching via an inverted microscope with a magnification of 40×. The areas of 8 images in each group were quantified with Image J 19.

**3D spheroid assay.** Fluorescently labeled EO771 mammary tumor cells were prepared by culturing them with green and red fluorescent dyes (4705 and 4706, respectively; Sartorius, Gottingen, Germany) for 20 min at 37°C. Approximately 1×10^4^ cells were cultured in a U-bottom low-adhesion 96-well plate (MS-9096UZ, S-Bio, Hudson, NH, USA) in complete DMEM (10% FBS, 1% antibiotics) or iTS CM. Four spheroid images for each group were captured with a fluorescence microscope (100× magnification, Olympus), and the cross-sectional area of fluorescently labeled spheroid was captured at 0 and 72 h and evaluated with Image J 20.

**Western blot analysis and immunoprecipitation.** Cells were lysed in a radio-immunoprecipitation assay buffer with protease inhibitors (PIA32963, Thermo Fisher Scientific) and phosphatase inhibitors (2006643, Calbiochem, Billerica, MA, USA). After cell lysis, proteins were fractionated by 10-15% SDS gels and electro-transferred to polyvinylidene difluoride transfer membranes (IPVH00010, Millipore, Billerica, MA, USA). After blocking 1 h with a blocking buffer (1706404, Bio-Rad, Hercules, CA, USA), the membrane was incubated overnight with primary antibodies and then with secondary antibodies conjugated with horseradish peroxidase for 45 min (7074S/7076S, Cell Signaling, Danvers, MA, USA). We employed antibodies against β-catenin (9562), cleaved caspase 3 (9661), caspase 3 (9662), CD44 (37259), Eno1 (3810), PD-L1 (29122), Runx2 (8486), Snail (3879) (Cell Signaling), MMP9 (sc-393859, Santa Cruz, Dallas, TX, USA), TRAIL (NB500-220, Novus, Centennial, CO, USA), CD95 (MA1-7622), p53 (MA5-12557, Invitrogen, Carlsbad, CA, USA), Ubc (PA5-76144, Thermo Fisher Scientific), and β-actin as a control (A5441, Sigma). The level of proteins was determined using a SuperSignal west femto maximum sensitivity substrate (PI34096, Thermo Fisher Scientific), and a luminescent image analyzer (LAS-3000, Fuji Film, Tokyo, Japan) was used to quantify signal intensities 21. The expression levels of Ubc and Eno1 in CM were detected by ELISA (MyBioSource).

For immunoprecipitation, protein samples were incubated with the agarose beads conjugated with protein A and rabbit IgG. They were then immunoprecipitated overnight with and without the beads conjugated with anti-Eno1 antibodies. The beads were isolated by centrifugation, washed and resuspended in PBS. To detect co-immunoprecipitated proteins, Western blotting was conducted using antibodies against Eno1 and CD44.

**Transfection.** EO771 and other cells were transfected with plasmids for β-catenin (31785, Addgene, Watertown, MA, USA), Wnt1 (35905, Addgene), FZD7 (159626, Addgene), and Lrp5 (115907, Addgene) while blank plasmids (FLAG-HA-pcDNA3.1; Addgene) were used as a control. Cells were grown in a 10-cm plate and transfected using lipofectamine®3000 (L300015, Thermo Fisher Scientific). The transfection reagents and DNA were mixed in two steps. In the first step, plasmids were diluted in 200 μL Opti-MEM (31985070, Thermo Fisher Scientific) and P3000 was added at the ratio of 2 μL transfection reagent to 1 μg DNA. In the second step, 20 μL Lipofectamine3000 was mixed with 200 μL Opti-MEM. These two mixtures were incubated at room temperature and the transfection was performed overnight. RNA interference with specific siRNAs was conducted to silence Eno1 (s234544), Ubc (s232597), Trail (4390771, Life Technologies, Carlsbad, CA, USA), and p53 (MBS8239623, MyBioSource), together with nonspecific negative control siRNAs (Silencer Select #1, Life Technologies; On-target Plus Non-targeting Pool, Dharmacon, Lafayette, CO, USA). Cells were transiently transfected with siRNA with Lipofectamine RNAiMAX (13778075, Life Technologies), and the medium was replaced by a regular culture medium after 24 h. The efficiency of silencing was assessed with immunoblotting 24 h after transfection.

**Preparation of conditioned medium (CM).** To generate tumor-suppressive CM, 2×10^6^ cells were transfected with β-catenin plasmids (40 ng/μL). Cells were grown in a 9 mL culture medium with antibiotics and a fraction of FBS consisting of factors smaller than 3 kDa. After 24 h, the medium was ultra-centrifuged (XL-90 ultracentrifuge; Beckman, Brea, CA, USA) at 100,000 g for 2 h to remove exosomes 22 and condensed 10-fold by the filtration (UFC900324, Amicon, Sigma) with a cut-off at 3 kDa.

***Ex vivo* tissue assay.** The usage of human breast cancer and prostate cancer tissues was approved by the Indiana University Institutional Review Board. Two human breast cancer tissues (~1 g each; estrogen receptor-positive and -negative) and prostate cancer tissue (~1 g), received from Simon Cancer Center Tissue Procurement Core, were manually fragmented with a scalpel into small pieces (0.5 ~ 0.8 mm in length). These pieces were grown in DMEM with 10% fetal bovine serum and antibiotics for a day. iTS cell-derived CM, which was treated with BML284 (10 μM, SC-222416, Santa Cruz) or transfected with β-catenin plasmids, was then added for three additional days, and a change in the fragment size was determined.

**Animal models.** The experimental procedures using animals were approved by the Indiana University Animal Care and Use Committee and were complied with the Guiding Principles in the Care and Use of Animals endorsed by the American Physiological Society. Mice were housed five per cage and provided with mouse chow and water ad libitum. In the mouse model of mammary tumors, C57BL/6 female mice (~8 weeks, Envigo, Indianapolis, IN, USA) were randomly assigned into five groups (14 mice per group). Each group received a subcutaneous injection of EO771 cells (3.0×10^5^ cells in 50 μL PBS) to the mammary fat pad on day 0. The placebo groups received cells transfected with a control vector, while two negative control groups received β-catenin-overexpressing cells or BML-treated cells. The two treatment groups received β-catenin overexpressing iTS CM or BML-treated iTS CM daily from day 1 to day 18. In the mouse model of tibial osteolysis, C57BL/6 female mice (5 groups as in the mouse model of mammary tumors; 8 mice per group) received an intra-tibial injection of EO771 cells (2.5×10^5^ cells in 20 μL PBS) to the right tibia on day 0. The two treatment groups received iTS cell-derived CM or BML-treated iTS CM as an injection into the intraperitoneal cavity daily from day 1. All animals were sacrificed on day 18 and tumor growth in the mammary fat pad and osteolysis in the tibia were determined.

To evaluate the effects of CMs for tumor invasion, the *in vivo* extravasation assay was conducted using C57BL/6 female mice (3 groups, 6 mice per group). EO771 cells were labeled with a green fluorescent dye and injected. Three groups included the placebo, the injection of β-catenin-overexpressing cells, and the injection of β-catenin overexpressing iTS CM via a lateral tail vein. Mice were sacrificed after 48 h for histological identification of extravascular tumor cells in the lung.

**μCT imaging and histology**. The tibia was harvested for μCT imaging using Skyscan 1172 (Bruker-MicroCT, Kontich, Belgium) and histology. Using the manufacturer-provided software, CT scans were performed with a pixel size of 8.99 μm and the captured images were reconstructed (nRecon v1.6.9.18) and analyzed (CTan v1.13). In histology, H&E staining was conducted as described previously 20, and immunohistochemistry was performed using the procedure previously described 23. The samples were analyzed in a blinded fashion.

**Pan-cancer survival analysis.** Using TCGA (The Cancer Genome Atlas) data, the pan-cancer survival analysis was conducted. We employed 9,880 primary tumor samples from 32 types of cancers from the UCSC Xena browser 24. Based on the observed effects of iTSC CMs, we focused on three protumorigenic genes, including MMP9, RUNX2, and Snail. The high expression group presented the elevated transcript level of these three genes above the median value, while the low expression group below the median value. Specifically, the high expression was defined as the patient with MMP9 expression value ≥ 9.02, RUNX2 expression ≥ 7.59, and Snail expression ≥ 6.37, whereas the low expression was defined as the patient with MMP9 expression value < 9.02, RUNX2 expression < 7.59, and Snail expression < 6.37. The Kaplan-Meier curve and log-rank test were used to evaluate survival probabilities with the survival package in R (v3.6.3).

**Whole-genome proteomics analysis**. Proteins in CM were analyzed in the Dionex UltiMate 3000 RSLC nano-system combined with the Q-exactive high-field hybrid quadrupole orbitrap mass spectrometer (Thermo Fisher Scientific). Proteins were first digested on beads using trypsin/LysC as described previously 25, 26 except digestion was performed in 50 mM ammonium bicarbonate buffer instead of urea. Digested peptides were then desalted using mini spin C18 spin columns (1910-050, The Nest Group, Southborough, MA, USA) and separated using a trap and 50-cm analytical columns 25, 27. Raw data were processed using MaxQuant (v1.6.3.3) 28 against the Uniprot mouse protein database at a 1% false discovery rate allowing up to 2 missed cleavages. MS/MS counts were used for relative protein quantitation. Proteins identified with at least 1 unique peptide and 2 MS/MS counts were considered for the final analysis.

To evaluate the tumor-suppressing capability of the predicted candidates, we employed fifteen recombinant proteins, including Hsp90ab1 (OPCA05157, Aviva System Biology, San Diego, CA, USA), Hspa8, Actg1, and Vim (NBP1-30278, H00000071-P01, NBP2-35139; Novus), Ubc, Ncl, Flna, Ppia, Aldoa, Eno1, Calm1, Lmna, Pkm, Eef2, and Ywhaz (MBS2029484, MBS146265, MBS962910, MBS286137, MBS8248528, MBS2009113, MBS2018713, MBS143846, MBS8249600, MBS1213669, and MBS143242; MyBioSource). In the MTT assay, 5 μg/mL of each of these recombinant proteins were added and the metabolic activity of tumor cells was evaluated.

**Statistical analysis.** For cell-based experiments, three or four independent experiments were conducted and data were expressed as mean ± S.D. In animal experiments, the sample size in the mouse model was chosen to achieve a power of 80% with p < 0.05. The primary experimental outcome was tumor weight for the mammary fat pad experiment and the bone volume ratio (BV/TV) for the tibia experiment. The secondary experimental outcome was tumor size for the mammary fat pad experiment and the trabecular number (Tb.n) for the tibia experiment. Statistical significance was evaluated using a one-way analysis of variance (ANOVA). Post hoc statistical comparisons with control groups were performed using Bonferroni correction with statistical significance at p < 0.05. The single, double, and triple asterisks in the figures indicate p < 0.05, 0.01, and 0.0001, respectively.

## Results

### The overexpression of β-catenin generated iTS cells

**Figure 1:**
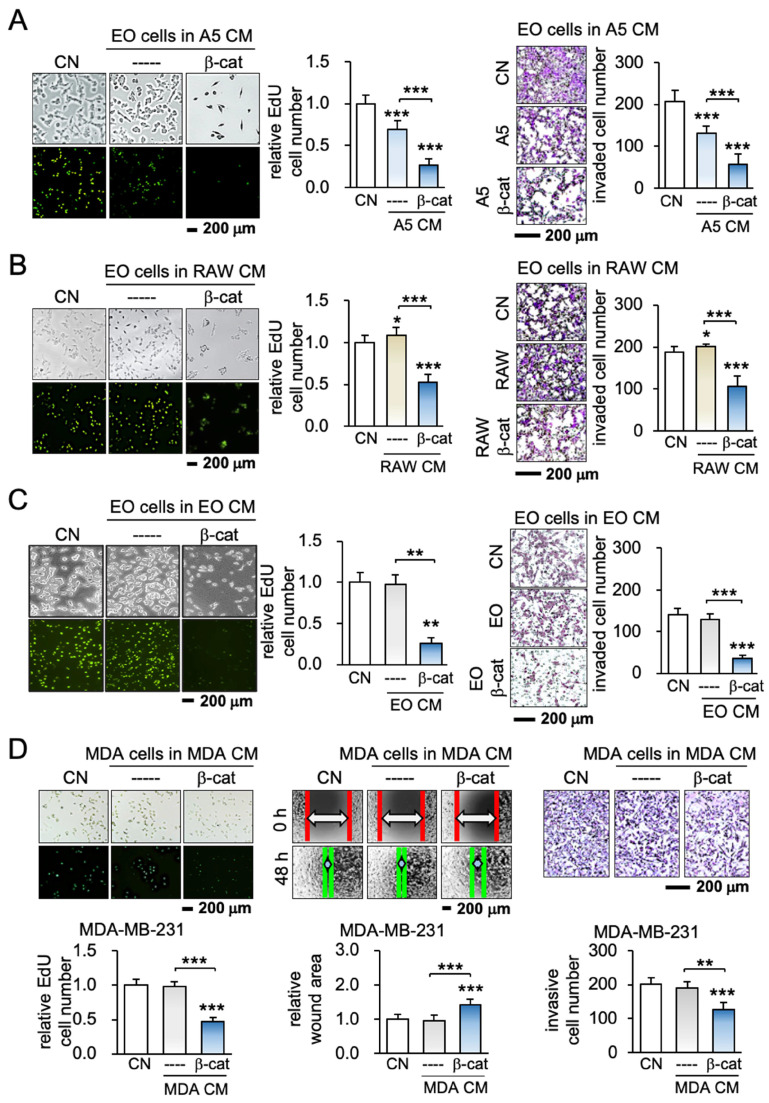
Generation of iTS cells from osteocytes, osteoclasts, and mammary tumor cells. CM = conditioned medium, CN = control (no CM treatment), β-cat = β-catenin plasmids, A5 = MLO-A5 osteocytes, RAW = RAW 264.7 osteoclasts, EO = EO771 mammary tumor cells, and MDA = MDA-MB-231 breast cancer cells. The single, double, and triple asterisks indicate p < 0.05, p < 0.01, and p < 0.0001, respectively. (A-C) Reduction in EdU-based proliferation and transwell invasion of EO771 mammary tumor cells by A5 osteocyte-derived, RAW264.7 osteoclast-derived, and EO771 breast cancer-derived iTS CMs in 2 days. These cells were transfected with β-catenin plasmids. (D) Inhibition of EdU-based proliferation, scratch-based migration, and transwell invasion of MDA-MB-231 breast cancer cells by β-catenin-overexpressing MDA-MB-231-derived CM in 2 days.

To test the effect of the overexpression of β-catenin, its constitutively active plasmids were transfected into MLO-A5 osteocytes, RAW264.7 osteoclasts, and EO771 mammary tumor cells. When EO771 cells were cultured for 2 days in each of the three β-catenin-overexpressing cell-derived CMs, EdU-based proliferation, scratch-based migration, transwell invasion, and the growth of 3-dimensional spheroids of mammary tumor cells were significantly reduced regardless of the source of CMs (Figure 1A-C; Figure S1). The result provided the first evidence that the overexpression of β-catenin generated iTS cells from varying cells including osteocytes, osteoclasts, and mammary tumor cells and their CMs presented anti-tumorigenic abilities.

### Cancer cells could become iTS cells

To further examine the possibility of generating iTS cells from other types of cancer cells, β-catenin was overexpressed in human and mouse cancer cell lines originating from cancers in the breast, pancreas, and prostate. The result revealed that iTS cancer cell-derived CM was able to inhibit the proliferation and invasion of their starting cancer cells as well as other cancer cells. For instance, MDA-MB-231 breast cancer cell-derived CM inhibited EdU-based proliferation, scratch-based migration, and transwell invasion of MDA-MB-231 breast cancer cells (Figure 1D). The same responses were observed when 4T1.2 mammary, TRAMP prostate PANC-1 pancreatic, and PC-3 prostate tumor cells were incubated with CM (Figure S2). Each of the iTS CMs from the six selected cancer cell lines strikingly suppressed EdU-based proliferation, and transwell invasion of the five non-self-cancer cells (Figure S3). Collectively, iTS cells and anti-tumor iTS CM could be generated by the overexpression of β-catenin in non-cancer cells as well as breast, prostate, and pancreatic cancer cells.

### iTS cell-derived CM inhibited the growth of cancer tissue fragments

**Figure 2:**
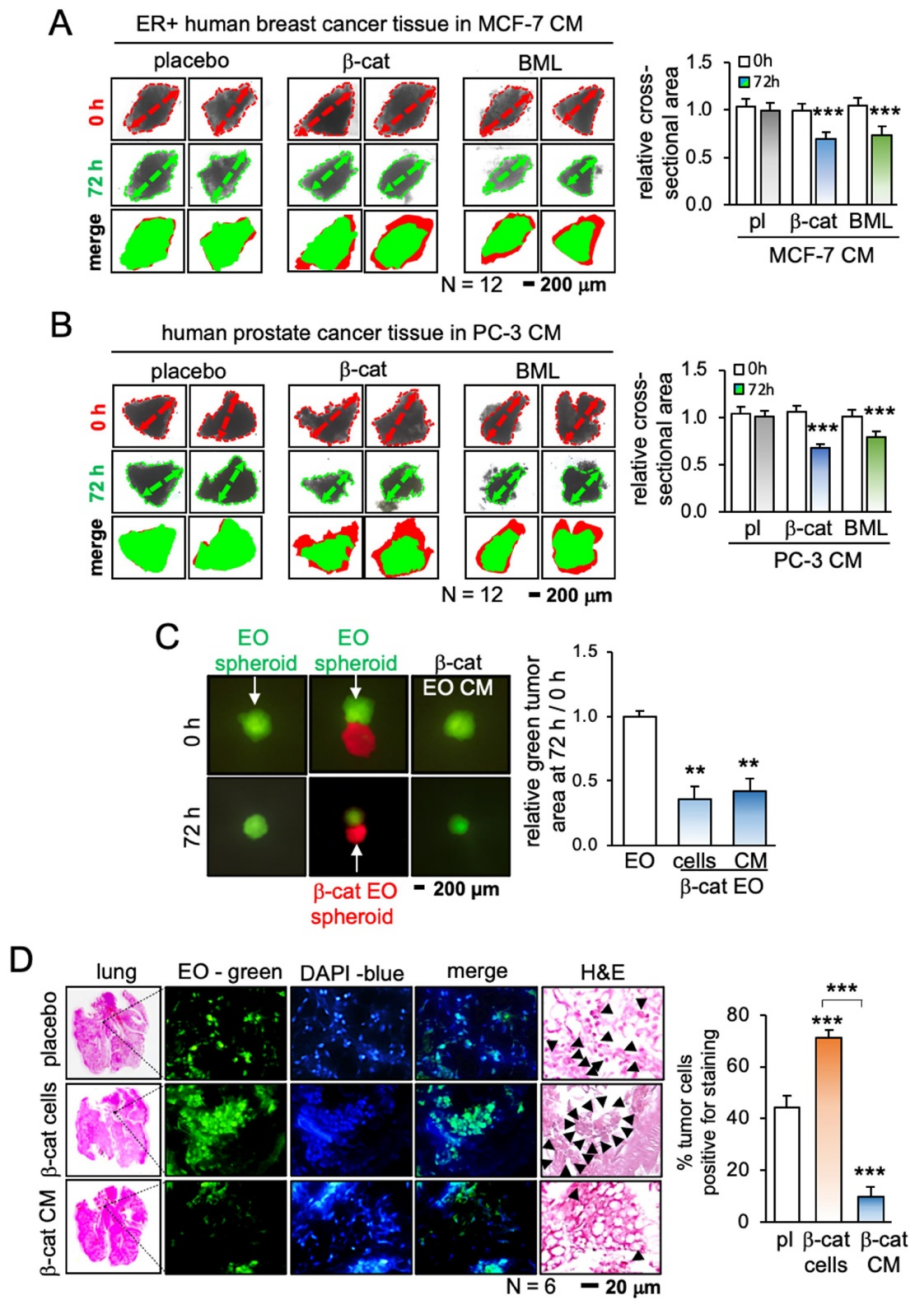
Inhibition of the growth of cancer tissue fragments and the tumor invasion to the lung by tissue-derived iTS CM. CM = conditioned medium, pl = placebo, β-cat = β-catenin plasmids, and EO = EO771 mammary tumor cells. The double and triple asterisk indicates p < 0.01 and 0.0001, respectively. (A) Shrinkage of breast cancer tissue fragments by β-catenin-overexpressing and BML284-treated MCF7-derived iTS CM in 72 h. (B) Shrinkage of prostate cancer tissue fragments by β-catenin-overexpressing and BML284-treated PC3-derived iTS CM in 72 h. (C) Shrinkage of EO771 mammary tumor spheroid (green) by co-culturing with β-catenin-overexpressing EO771 spheroid (red) and by β-catenin-overexpressing EO771-derived iTS CM in 72 h. (D) Extravasation assay for examining the invasion of tumor cells into the lung. Increase in the invaded cells by inoculating β-catenin-overexpressing EO771 cells, and a decrease by the administration of β-catenin-overexpressing EO771 cell-derived CM. The green fluorescently labeled EO771 cells were injected into the tail vein and mice were sacrificed in 48 h for histological inspection. The black arrows indicate tumor cells.

Having shown the anti-tumor capability of iTS CM, we next evaluated the efficacy in tumor suppression using 3 freshly isolated human cancer tissues from patients with breast cancer (estrogen receptor-positive and -negative) and prostate cancer. CM was prepared from the cancer cells by transfecting β-catenin plasmids or applying 10 μM of BML284, a pharmacological activator of Wnt signaling, for 1 day. Compared to the placebo CM, both CMs generated with β-catenin plasmids and BML284 significantly shrank the size of cancer tissue fragments in the *ex vivo* tissue assay (Figure 2A-B; Figure S4A). By contrast, the direct addition of BML284 to the fragments modestly increased their size, although this effect did not reach statistical significance (Figure S4B-D). In a 3-dimensional assay using a pair of tumor spheroids (red and green with and without β-catenin overexpression, respectively), the red β-catenin-overexpressing spheroid inhibited the growth of the green spheroids. Similarly, CM from β-catenin-overexpressing cells shrank the green control spheroids (Figure 2C).

### iTS CM inhibited the tumor invasion to the lung and the growth of mammary tumors

**Figure 3:**
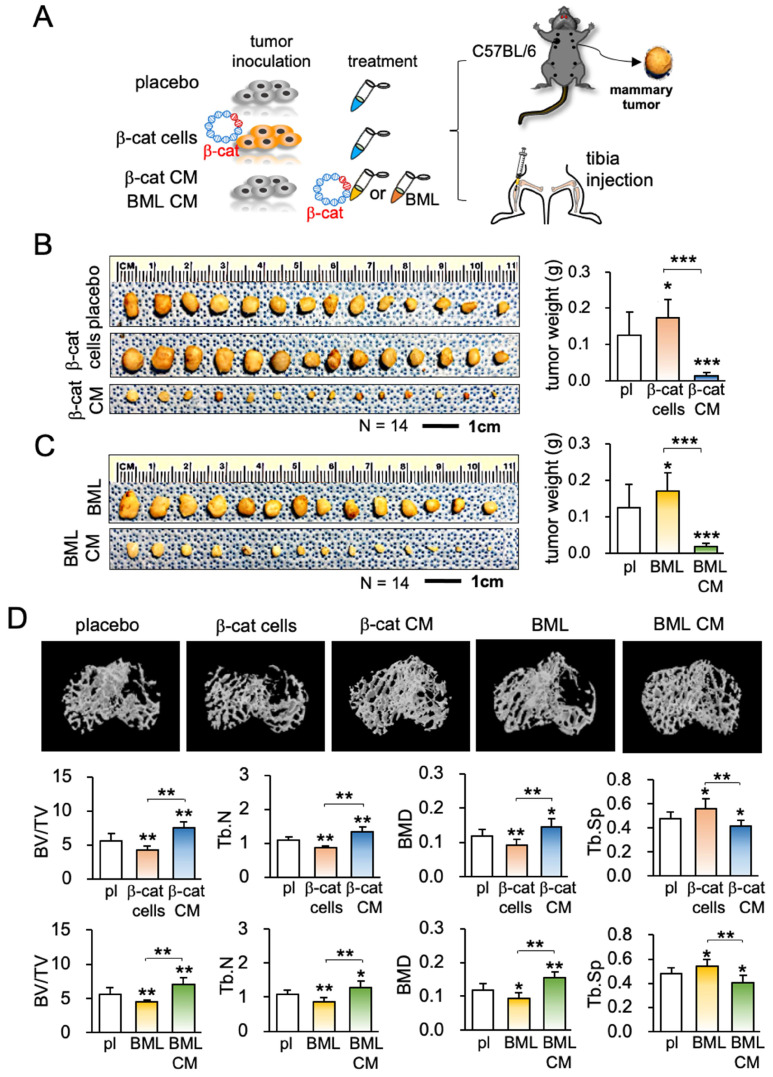
Inhibition of tumor growth and bone loss by iTS CM in the mouse model. CM = conditioned medium, pl = placebo, β-cat = β-catenin plasmids, and BML = BML284. The single, double, and triple asterisks indicate p < 0.05, p < 0.01, and p < 0.0001, respectively. (A) Procedure for the inoculation of EO771 cells to the mammary fat pad and tibia. (B) Increase in tumor weight by inoculating β-catenin-overexpressing EO771 cells, and a decrease by the administration of β-catenin-overexpressing EO771 cell-derived CM in 2 weeks (N = 10). (C) Increase in tumor weight by the systemic administration of BML284, and a decrease by the administration of BME284-treated EO771 cell-derived CM in 2 weeks (N = 10). (D) μCT images of the proximal tibia and BV/TV (bone volume ratio), BMD (bone mineral density), Tb.N (trabecular number), and Tb.Sp (trabecular separation) for 5 groups of C57BL/6 female mice (N = 10). They are the placebo (no treatment), inoculation of β-catenin-overexpressing cells (β-cat cells), administration of β-catenin-overexpressing EO771 cell-derived CM (β-catenin CM), inoculation of BML284-treated cells (BML), and administration of BML284-treated cell-derived CM (BML CM).

The anti-tumor capability of iTS CM has so far been tested *in vitro* and *ex vivo*. The efficacy of iTS CM was next examined in the mouse model. In the *in vivo* extravasation assay to evaluate the level of metastasis to the lung, EO771 mammary tumor cells were intravenously injected into the tail vein. Compared to the placebo group that received control CM, two iTS CM groups (β-catenin overexpression and BML284 pre-treatment) markedly reduced the number of tumor cells in the lung in 2 days (Figure 2D; Figure S5A). In the mouse model of mammary tumors, a daily intravenous injection of iTS CM (β-catenin overexpression and BML284 pre-treatment) for 2 weeks significantly reduced the size and weight of mammary tumors (Figure 3A-C). By contrast, the direct inoculation of β-catenin-overexpressing EO771 tumor cells stimulated the growth of mammary tumors. Of note, the average body weight did not significantly change during the treatment with iTS CM (Figure S5B).

### iTS CM inhibited the tumor progression and osteolysis

Because of a frequent metastasis to the bone from breast cancer, our next examination was the effect on the tumor-invaded bone. We produced iTS CM using EO771 mammary tumor cells. Compared to the placebo and two negative controls (injection of β-catenin-overexpressing EO771 cells and BML284-treated EO771 cells), μCT imaging of the tumor-inoculated tibia revealed that β-catenin-overexpressing iTS CM as well as BML284-treated iTS CM significantly reduced tumor-driven osteolysis (Figure 3D). In response to the iTS CM, the bone volume ratio (BV/TV), trabecular number (Tb.N), and bone mineral density (BMD) were elevated in the proximal tibia, while the trabecular separation (Tb.Sp), an indicator of trabecular bone loss, was reduced. These changes supported the ability of iTS CM to protect against cancer-induced osteolysis. In contrast, the inoculation of β-catenin-overexpressing EO771 cells and BML284-treated EO771 cells stimulated bone loss by reducing BV/TV, Tb.N, BMD with an increase in Tb.Sp.

### Eno1 and Ubc were identified as tumor-suppressing factors

**Figure 4:**
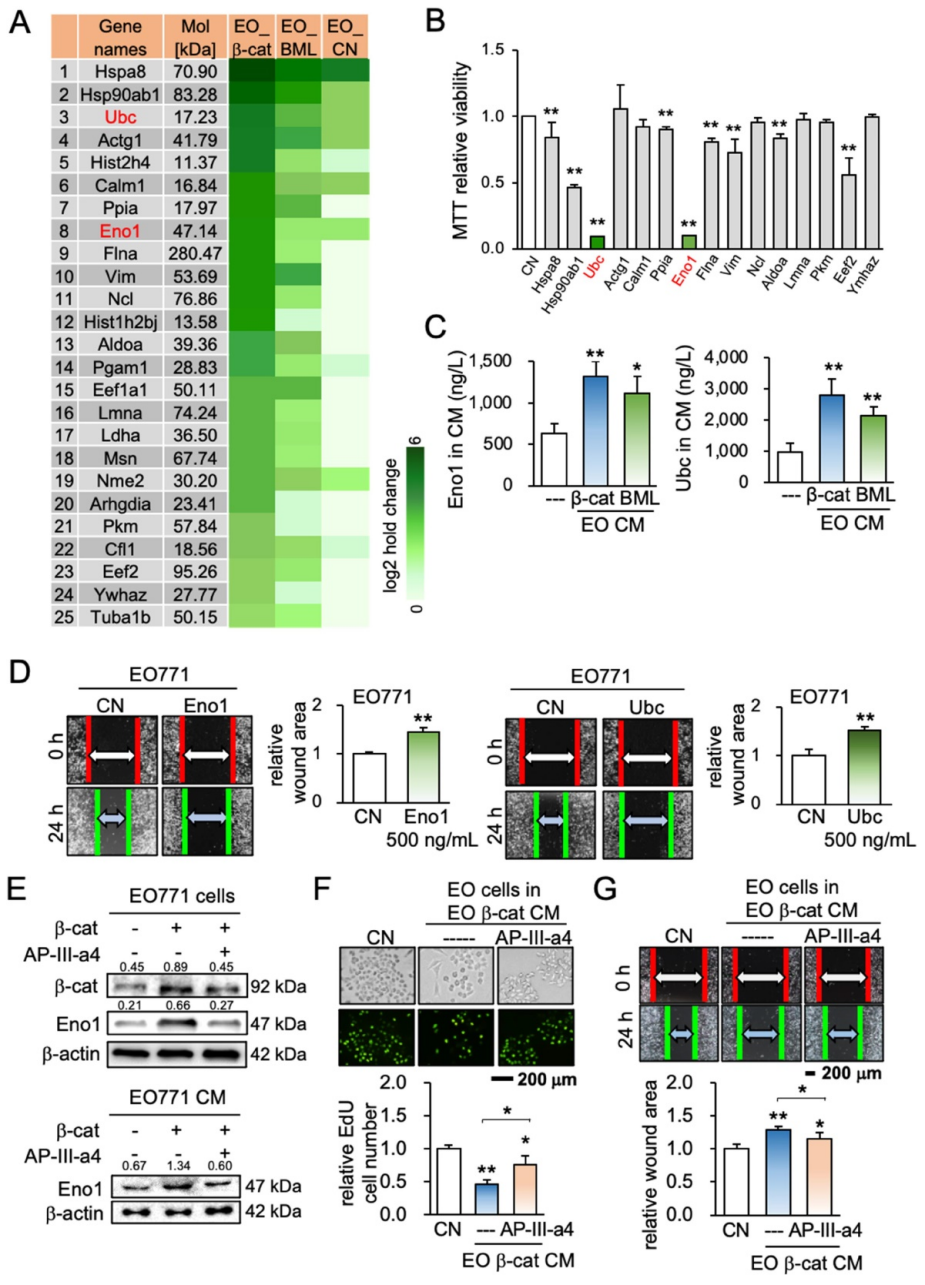
Mass spectrometry-based prediction of tumor suppressors and the effect of enolase 1 and ubiquitin C. EO = EO771 mammary tumor cells, CM = conditioned medium, CN = control (no CM treatment), β-cat = β-catenin plasmids, BML = BML284, Eno1 = Enolase 1, and Ubc = ubiquitin C. The single and double asterisks indicate p < 0.05 and p < 0.01, respectively. (A) List of 25 top tumor suppressor candidates identified by mass spectrometry-based proteomics analysis. (B) Reduction in MTT-based proliferation of EO771 mammary tumor cells by 9 recombinant proteins (5 μg/mL) in 48 h. (C) Levels of enolase 1 and ubiquitin C in β-catenin-overexpressing and BML284-treated EO771 CMs by ELISA. (D) Inhibition in the scratch-based migration of EO771 mammary tumor cells by enolase 1 and ubiquitin C in 24 h. (E) Expression of β-catenin and enolase 1 in EO771 cells that were treated with AP-Ⅲ-a4, an inhibitor of enolase 1. (F&G) Repressive effects of AP-Ⅲ-a4 on the proliferation (in 48 h) and migration of EO771 cells (in 24 h) by β-catenin overexpressing iTS CM.

To predict specific proteins that were enriched and responsible for the observed anti-tumor action, we conducted mass spectrometry-based proteomics. We identified 885 proteins in 4 CMs (medium control, CM control, β-catenin-overexpressing CM, and BML284-treated CM), in which 97 proteins were present in β-catenin-overexpressing EO771 CM. Eighty-nine proteins were expressed higher in β-catenin-overexpressing CM than the control CM, and 25 top candidates as potential tumor suppressors are listed (Figure 4A). Based on the availability of recombinant proteins, the effects of 15 proteins (5 μg/mL) on the viability of EO771 tumor cells were evaluated (Figure 4B). Among them, the administration of Eno1 and Ubc induced a notable decrease in the MMT-based viability. Hereafter, we mainly focused on the role of these two proteins.

**Figure 5:**
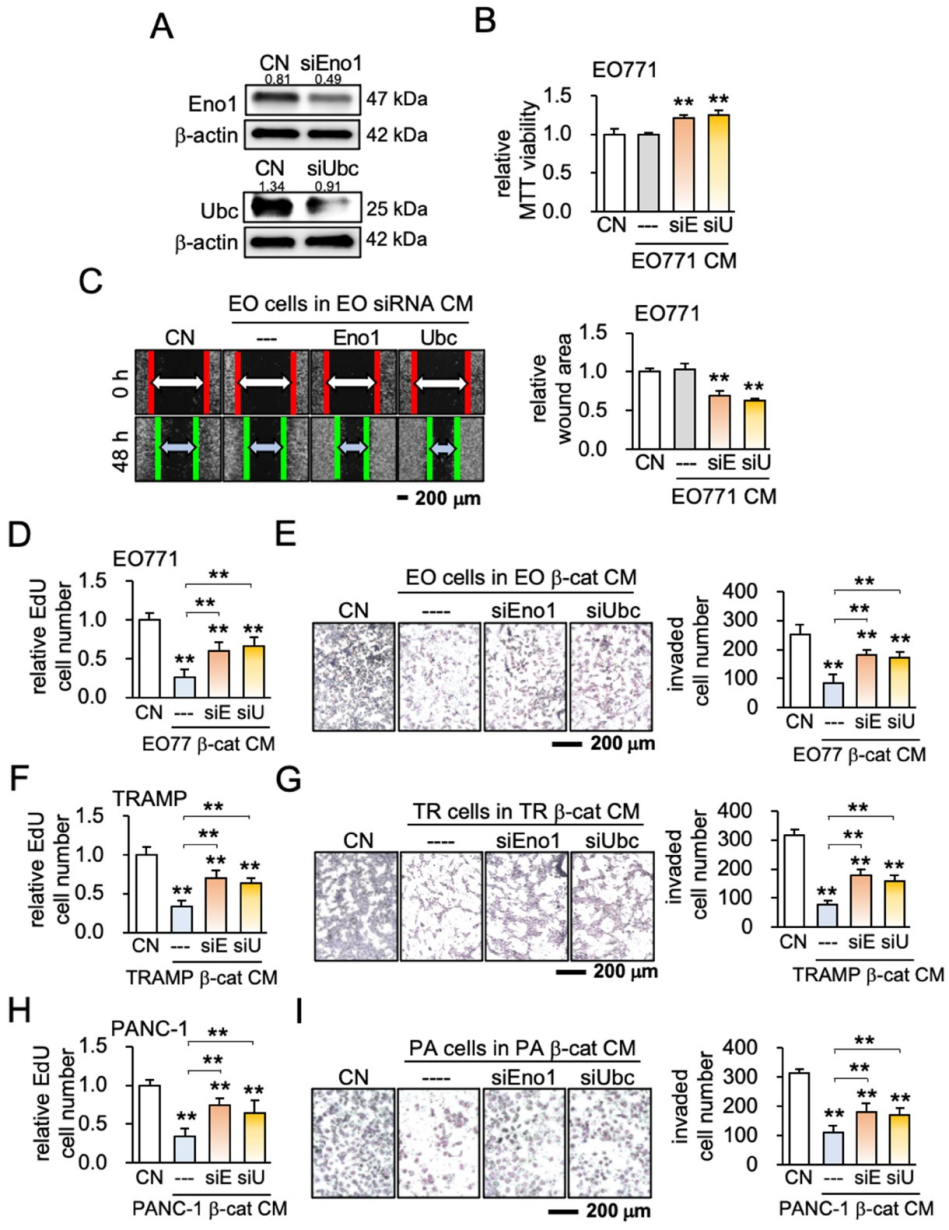
Effect of silencing enolase 1 and ubiquitin C. CM = conditioned medium, CN = control (no CM treatment), β-cat = β-catenin plasmids, siEno1 = Enolase 1 siRNA, siUbc = ubiquitin C siRNA, EO = EO771 mammary tumor cells, TR = TRAMP prostate cancer cells, and PA = PANC-1 pancreas cancer cells. The double asterisk indicates p < 0.01. (A) siRNA-mediated knockdown of enolase 1, and ubiquitin C in EO771 breast cancer cells. (B&C) Promotion of MTT-based proliferation, and scratch-based migration of EO771 breast cancer cells by enolase 1 and ubiquitin C siRNA-treated CMs in 2 days. (D-I) Effects of enolase 1 and ubiquitin C siRNAs. Silencing these two proteins significantly prevented the reduction in EdU-based proliferation and Transwell invasion of EO771, TRAMP, and PANC-1 cells by their own β-catenin-overexpressing iTS CMs in 2 days.

ELISA and Western blotting confirmed that the levels of Eno1 and Ubc were elevated in EO771 mammary tumor cell-derived iTS CM (Figure 4C, Figure S6A). Eno1 and Ubc inhibited the scratch-based migration of EO771 breast cancer (Figure 4D), as well as the proliferation and invasion of TRAMP prostate and PANC-1 pancreatic cancer cells (Figure S6B-C). The application of a pharmacological agent (ENOblock - AP-III-a4), an inhibitor of Eno1, suppressed the inhibitory effect of β-catenin-overexpressing iTS CM on the migration and invasion of mammary tumor cells (Figure 4E-G). Also, an inhibitor of ubiquitin E3 ligase, Pomalidomide, interfered with the inhibitory effect of β-catenin-overexpressing iTS CM on the proliferation and invasion of mammary tumor cells (Figure S6D-G). Furthermore, siRNA-mediated silencing of Eno1 and Ubc promoted the proliferation and migration of EO771 mammary tumor cells (Figure 5A-C) and blocked the inhibitory effects of β-catenin-overexpressing CM on mammary, prostate, and pancreatic tumor cells (Figure 5D-I). Taken together, multiple lines of evidence supported the anti-tumor action of Eno1 and Ubc in iTS CM.

### Tumor selective inhibition was achieved by conditioned medium, Eno1 and Ubc

**Figure 6:**
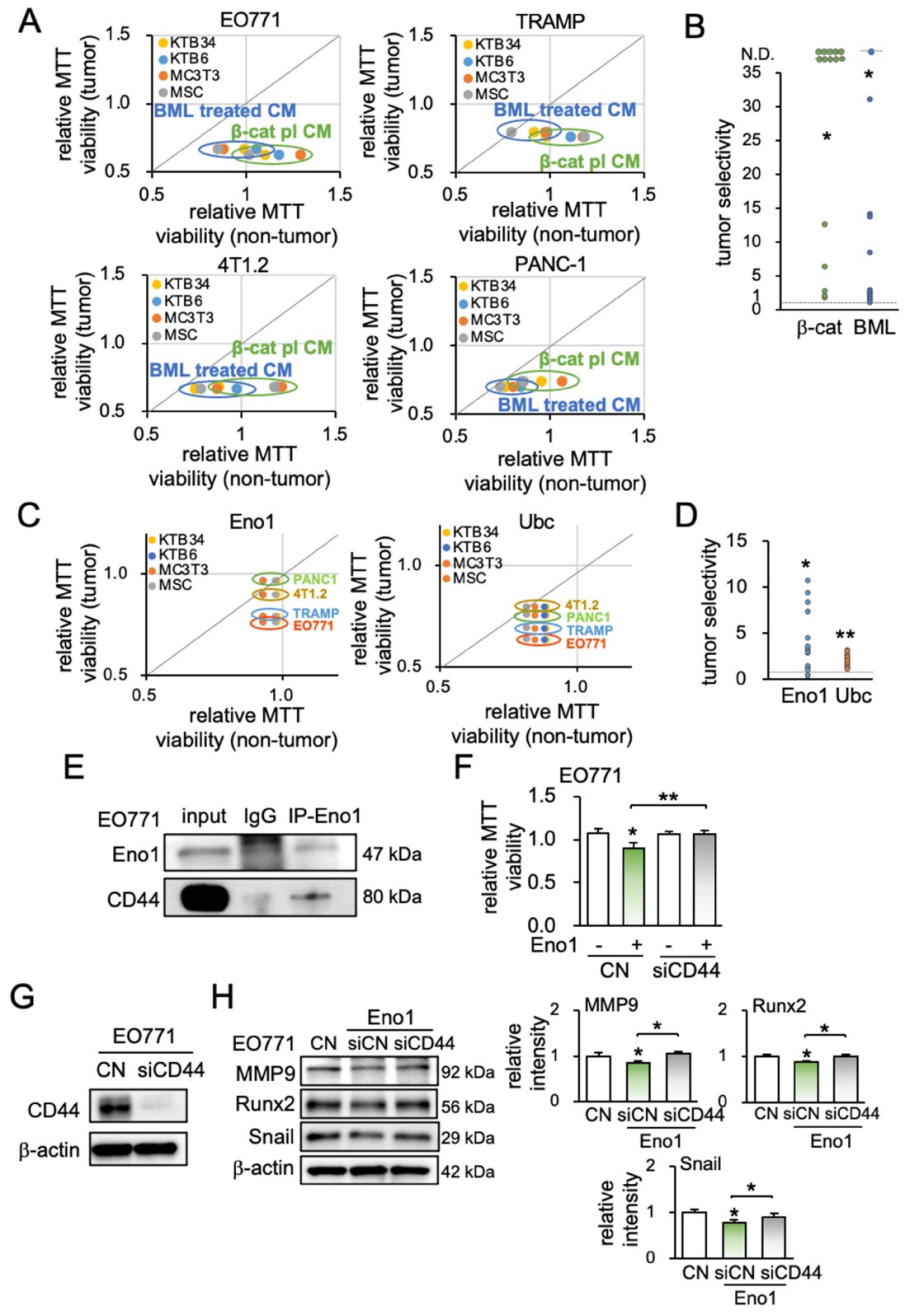
Tumor selectivity and the involvement of CD44. A5 = MLO-A5 osteocytes, β-cat = β-catenin plasmids, BML = BML284, Eno1 = Enolase 1, Ubc = ubiquitin C. The signal and double asterisk indicates p < 0.01 and p < 0.05, respectively. (A) Comparison of MTT-based viability of four non-tumor cells (KTB34, KTB6, MC3T3, MSC) and four tumor cells (EO771, TRAMP, 4T1.2, PANC-1) in response to β-catenin-iTS CM and BML-iTS CM in 2 days. (B) Tumor selectivity of EO771 CM, 4T1.2 CM, TRAMP CM, and PANC-1 CM. MTT-based tumor selectivity is defined as the ratio of (reduction in tumor cells) to (reduction in non-tumor cells). The selectivity value above 1 indicates that MTT-based inhibition is more selective to tumor cells than non-tumor cells. Of note, N.D. = not defined since the viability of non-tumor cells is stimulated. (C&D) Tumor selectivity of Enolase 1 and ubiquitin C. (E) CD44 was co-immunoprecipitated with Eno1. The protein extracts of EO771 cells were incubated with anti-Eno1 antibody using the protein A/G beads. Immunoprecipitates and total cell lysates were analyzed by Western blotting with anti-CD44 and anti-Eno1 antibodies as indicated. (F&G) siRNA knockdown of CD44 suppressed Eno1-mediated inhibition of the proliferation of EO771 cells. (H) siRNA knockdown of CD44 suppressed Eno1-mediated downregulation of MMP9, Runx2, and Snail in EO771 cells.

Ideally, CM should selectively inhibit tumor cells with few negative effects on non-tumor cells. We next examined whether iTS CM, Eno1, and Ubc may preferentially inhibit the progression of tumor cells. The tumor selectivity was defined as the MTT-based ratio of the reduction in the viability of tumor cells to that of non-tumor cells. The tumor selectivity larger than one indicates a favorable selection and we evaluate using four tumor cell lines (EO771, TRAMP, 4T1.2, and PANC-1) and four types of non-tumor cells (KTB34, KTB6, MSCs, and MC3T3 osteoblasts) (Figure 6A). The tumor selectivity of β-catenin-overexpressing iTS CM was above 1 in all 16 cases, while that of BML-iTS CM was above 1 in 15 out of 16 cases (Figure 6B; Figure S7A). In many cases, tumor selectivity was undefined (N.D.) in which CM stimulated the viability of non-tumor cells. We also detected the favorable tumor selectivity with Eno1 and Ubc (Figure 6C-D, Figure S7B).

### Eno1-CD44 regulatory axis was involved in the anti-tumor action of iTS CM

To assess the potential involvement of the Eno1-CD44 axis in the tumor-suppressive action of Eno1, we conducted an immunoprecipitation assay. CD44 is a transmembrane adhesion receptor and acts as a regulator of the Wnt receptor complex. The binding partner of Eno1 was isolated from EO771 protein extracts, and Western blotting revealed that CD44 was co-immunoprecipitated with Eno1 (Figure 6E). Notably, the anti-tumor effect of 1 μg/mL of recombinant Eno1 proteins on MMT-based viability was suppressed when CD44 was silenced (Figure 6F). Furthermore, the silencing of CD44 in EO771 cells suppressed Eno1-mediated downregulation of MMP9, Runx2, and Snail (Figure 6G-H).

### iTS CM, Eno1, and Ubc downregulated tumor-promoting genes and PD-L1

**Figure 7:**
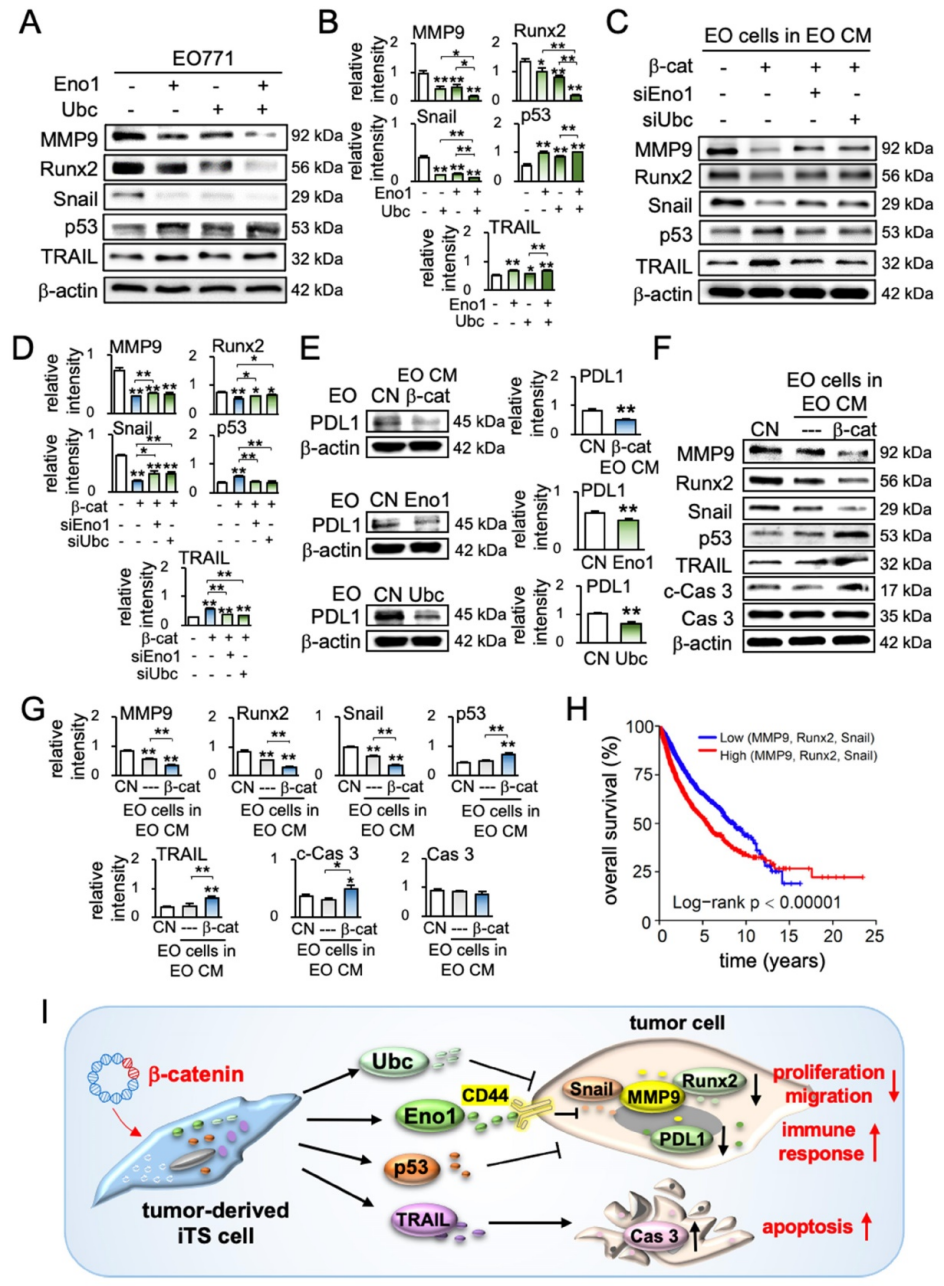
Effects of enolase 1, ubiquitin C, and iTS CM on the expression of tumor-promoting and tumor-suppressing genes. CM = conditioned medium, CN = control (no CM treatment), β-cat = β-catenin plasmids, siEno1 = Enolase 1 siRNA, siUbc = ubiquitin C siRNA, EO = EO771 mammary tumor cells. (A&B) Expression of MMP9, Runx2, Snail, p53, and TRAIL in response to enolase 1 and ubiquitin C in EO771 breast cancer cells. (C&D) Expression of MMP9, Runx2, Snail, p53, and TRAIL in response to β-catenin-overexpressing iTS CM impaired by siRNAs specific to enolase 1 and ubiquitin C. (E) Expression of PDL1 in EO771 mammary tumor cells in response to β-catenin-overexpressing iTS CM, enolase 1, and ubiquitin C. (F&G) Expression of MMP9, Runx2, Snail, p53, TRAIL, and caspase 3 in EO771 mammary tumor cells in response to β-catenin-overexpressing pre-treatment tumor cell-derived CM. (H) Low survival for cancer patients with a high transcript level of MMP9, Runx2, or Snail. (I) Proposed regulatory mechanism to inhibit tumor progression by iTS-CM. According to the mechanism, β-catenin-overexpressing iTS cells secrete ubiquitin C (Ubc), enolase 1 (Eno1), p53, and Trail. They suppress the progression of tumor cells by downregulating MMP9, Runx2, Snail, and PDL1, while upregulating cleaved-caspase 3. It should be noted that Eno1 interacts with CD44 and inhibits MMP9, Runx2, and Snail.

To further understand the anti-tumor action of iTS CM, we examined the expression of protumorigenic proteins such as MMP9, Runx2, and Snail, as well as a tumor-suppressing protein, p53, and an apoptosis-inducing factor, TRAIL. Western blot analysis revealed that Eno1, Ubc, and their combined application reduced MMP9, Runx2, and Snail, and elevated p53 and TRAIL (Figure 7A-B). Consistently, the inhibition of Eno1 reversed the responses (Figure S8A). Furthermore, the pharmacological inhibitors impaired the effect of β-catenin-overexpressing iTS CM on the expression of MMP9, Runx2, Snail, p53, and TRAIL (Figure S8B). Similarly, the siRNA-mediated knockdown of Eno1 and Ubc repressed the effect of β-catenin-overexpressing iTS-CM (Figure 7C-D). Also, their silencing resulted in a decrease in p53 and TRAIL in both EO771 cells and EO771-derived CM (Figure S8C-D). Besides the regulation of the selected protumorigenic genes, we examined the potential role of iTS CM in immune responses. In particular, we focused on the expression of PD-L1, which is an immune escape modulator in tumor cells and its inhibition is a major strategy in immunotherapy. We observed that the application of β-catenin-overexpressing iTS CM, Eno1, and Ubc significantly reduced the level of PD-L1 in EO771 mammary tumor cells as well as TRAMP prostate tumor cells (Figure 7E, Figure S8E).

### iTS CM regulated pro- and anti-tumorigenic genes for improving survival

We have shown that iTS CM can reduce protumorigenic genes and elevate anti-tumorigenic genes. For instance, EO771 mammary tumor-derived iTS CM increased the levels of p53 and TRAIL (Figure S9A). Besides the overexpression of β-catenin, we observed that the overexpression of p53 in EO771 cells generated iTS cells and their CM markedly inhibited the expression of MMP9, Runx2, and Snail in EO771 cells (Figure S9B). Consistently, the silencing of p53 reversed the responses (Figure S9C). Furthermore, the treatment with 400 μg/mL TRAIL recombinant proteins increased the level of cleaved caspase 3 (Figure S9D), and its RNA interference suppressed the elevation (Figure S9E). In response to EO771-derived iTS-CM by β-catenin overexpression and BML284 pre-treatment, EO771 cells downregulated MMP9, Runx2, and Snail and elevated p53, TRAIL, and cleaved caspase 3 (Figure 7F-G, Figure S9F-G). By contrast, the overexpression of β-catenin in EO771 cells increased the levels of MMP9, Runx2, and Snail (Figure S9H).

This study has examined so far the role of β-catenin and BML284 in generating iTS CM from tumor cells as well as non-tumor cells, whereas our previous study showed the same role of Lrp5, a Wnt co-receptor, in producing anti-tumor CM from osteocytes and MSCs. Not all genes in Wnt signaling can create iTS CM, and the negative control genes in Wnt signaling included Wnt1 and Fzd7. The overexpression of Wnt1 and Fzd7 in EO771 cells did not increase the level of β-catenin and did not reduce the MMT-based viability of EO771 cells (Figure S10A&B). While Lrp5-overexpressing CM elevated the level of CD95, an apoptosis-linked gene, in EO771 cells, Wnt1- and Fzd7-overexpressing CM decreased the level of CD95 and did not affect the levels of MMP9, Runx2, and Snail in EO771 tumor cells (Figure S10C&D).

In summary, we conducted a pan-cancer survival analysis, including 9,676 primary tissue samples from 32 types of cancers. To assess the possible impact of the application of iTS CM, we evaluated the value of the downregulation of MMP9, Runx2, and Snail. The result revealed that the low expression group (N = 2,198), which had a lower transcript level of MMP9, Runx2, or Snail than the median value, presented a significantly favorable survival outcome compared to the high expression counterpart (N = 2,217) (*p* < 0.00001, Figure 7H). The detailed information on TCGA database for pan-cancer gene expressions and the number of samples were shown in Table S1.

## Discussion

We presented in this study that the overexpression of β-catenin in tumor cells can generate iTS cells and their CM exhibits an anti-tumor capability. The treatment of tumor cells by BML284, an activator of Wnt signaling, also bestowed an anti-tumor capability to tumor cell-derived CM. By contrast, the inoculation of β-catenin-overexpressing tumor cells to the mammary fat pad or the administration of BML284-treated cells on day 1 significantly elevated the growth of mammary tumors and tumor-driven bone loss. Collectively, the result demonstrates that the activation of β-catenin-mediated Wnt signaling is a double-edged sword. Its activation in tumor cells made tumor cells proliferative and migratory, whereas tumor cell-derived CM acted as an anti-tumor agent. This contrasting effect was also observed in the *ex vivo* cancer tissue assay, in which BML284-treated tumor-derived CM inhibited the growth of the tumor fragments of the same origin.

Protein analysis of tumor-cell-derived CM revealed that the overexpression of β-catenin downregulated tumor-promoting genes such as MMP9 30, Runx2 31, and Snail 32, 33, and upregulated a tumor-suppressing gene, p53 34, 35 as well as an apoptosis-inducing factor, TRAIL 36, 37. Of note, p53 is reported to be present in the blood circulation and the circulating p53 affects tumor growth 38, 39, whereas TRAIL is secreted in lipid microvesicles and induces apoptosis of cancer cells 40, 41. The unexpected result from the mass spectrometry-based proteomics analysis was the enrichment of extracellular Eno1 and Ubc in β-catenin-overexpressing CM and their beneficial role in the upregulation of p53 and TRAIL in tumor cells (Figure 7I).

As the activation of Wnt signaling is paradoxical for the therapeutic strategy, so as the predicted tumor suppressors such as Eno1 and Ubc are. Eno1 is a glycolytic enzyme that catalyzes the conversion of 2-phosphoglycerate to phosphoenolpyruvate, while Ubc is a key regulator in ubiquitination that degrades unneeded or damaged proteins 42. Eno1 is mostly located in the cytoplasm and its elevated expression and tumor-promoting functions are reported in many cancer tissues including the liver, colon, and lung 43, 44. Eno1 is also reported to act as a cell surface receptor and contributes to activating immune responses 45. In this study, we identified CD44 as a binding partner of Eno1 and observed CD44-mediated inhibition of Eno1's anti-tumor action. CD44 is known to have a dual role in breast cancer progression and its action may depend on its interacting partners 11. Ubiquitin is in general considered oncogenic 46, 47. Its high expression is reported in many cancers including ovarian cancer, glioma, and gastric cancer, and its inhibition is shown to suppress the progression of tumors 48. The result of this study indicates that the tasks of intracellular and extracellular Eno1 and Ubc can be different. One such multi-tasking protein is histone H4 that packs DNA in the nucleus and stimulates innate immune responses outside of the cell 49. We previously reported that extracellular histone H4 served as a tumor suppressor 50. Another moonlighting example is a high mobility group box 1 (HMGB1) protein. Extracellular HMGB1 acts as a pro-tumor protein due to its role with cytokines and chemokines, whereas intracellular HMGB1 acts as an anti-tumor protein due to its ability to sustain genome stability 51.

Since exosomes in CM can act as tumor-promoting agents 52, we removed exosomes by ultra-centrifugation 53, 54. Without ultra-centrifugation, however, iTS CM showed tumor-suppressing capability (data not shown). While we observed beneficial effects of iTS CM, further studies can examine any dependence of the originating iTS cell types on the targeted tumor types. We showed that the anti-tumor effects can be induced not only by the overexpression of β-catenin and Lrp5, a Wnt co-receptor, but also by the administration of the tumor-promoting chemical compound, BML284. BML284 promotes tumors if directly administered to mice but suppresses tumors if used indirectly as an agent to generate iTS cells. It is of interest whether other signaling pathways, such as PI3K 55, 56 and NFκB 57, 58 may also induce iTS cells from tumor cells. In Wnt signaling, we observed that the overexpression of Wnt1 and Fzd7 did not generate iTS cells. In the generation of iTS cells from osteocytes, we have previously shown that the overexpression of β-catenin and Lrp5 but not Lrp6 promoted the anti-tumor capabilities and the protection of tumor-invased bone 1. In osteocytes and MSCs, the overexpression of multiple pro-tumorigenic genes and the administration of tumor-promoting compounds have been shown to generate iTSC that significantly inhibited the progression of adjacent tumors 1-3. Further analyses are needed to elucidate the requirement for creating effective iTS CM from varing tumor and non-tumor cells, considering types of target cancer cells, as well as pathways and genes to be activated and overexpressed. In breast cancer, for instance, it is important to elucidate any dependence of the efficacy of tumor-suppressive secretomes on hormonal receptor status.

One rationale for building beneficial iTS cells can be linked to cell competition, which is an evolutionarily conserved mechanism. Selfish DNA is a term used to describe the efficient replicators that multiply at the expense of less efficient competitors 59, 60. Tumor cells might also be viewed as selfish beings and after sufficient time, aggressive tumor cells with high survival fitness may tend to dominate 61, 62. It is recommended to elucidate the potential linkage of the acquired tumor-suppressing ability to the natural selection process for tumor cells. The survival of the fittest tumor cells may cause the removal of less competitive tumor cells through interactions with the microenvironment 63, 64. This principle of tumor-tumor interaction can be the basis for the described cancer treatment strategy. We postulate that the property of β-catenin-overexpressing or BML284-treated CM is reminiscent of dominant clones, which may possess an advantage of actively killing the less-fit neighboring cells. We also showed that the expression of PD-L1, one of the major immune escape factors in tumor cells 13, was downregulated by iTS CM. Besides tumor-tumor interactions and tumor-bone interactions, the survival of tumor cells is linked to a complex network of interactions among many types of cells.

The result herein could shed light on the effects of surgical removal of primary cancers. Most clinical data suggest a favorable outcome with surgical extirpation, while in some cases accelerated tumor recurrence is reported 65. Concomitant tumor resistance supports the inhibition of the growth of secondary tumors by a tumor-bearing host 66. Potential reasons for the surgery-linked detrimental outcomes can be linked to trauma, inflammation, tumor-driven immune responses, the competition of nutrient acquisition, and tumor-driven tumor-suppressive secretory factors 66, 67. This study indicates that influential primary tumors may generate tumor-suppressive factors, which inhibit the growth and migration of less aggressive neighboring tumors. In summary, we demonstrated that the survival of tumor cells was impeded by their cohorts that overexpress a specific gene such as β-catenin in Wnt signaling. These tumor-derived iTS cells secreted anti-tumor factors such as Eno1 and Ubc in their CM and eliminate neighboring tumor cells. A pan-cancer survival analysis supported the notion that the complete or partial secretomes may offer a novel therapeutic option to treat primary cancers and prevent bone loss from metastasized breast cancer.

## Supplementary Material

Supplementary figures and table.

## Abbreviations

ANOVA: a one-way analysis of variance
BMD: bone mineral density
BV/TV: bone volume ratio
CM: conditioned medium
ER: estrogen receptor
FBS: fetal bovine serum
HMGB1: high mobility group box 1
iTSCs: induced tumor-suppressing cells
LAS: luminescent image analyzer
MSCs: mesenchymal stem cells
PD-L1: programmed death-ligand1
PVDF: polyvinylidene difluoride
Tb.n: trabecular number
and Tb.Sp: trabecular separation
