# Supplementary material for: Generation of the tumor-suppressive secretome from tumor cells
Source: Theranostics. 2021 Jul 25;11(17):8517–34. doi: 10.7150/thno.61006 (PMC8344019; doi:10.7150/thno.61006)
Supplement: Supplementary file 1 — Supplementary figures and table. [file thnov11p8517s1.pdf]

Supplementary Information

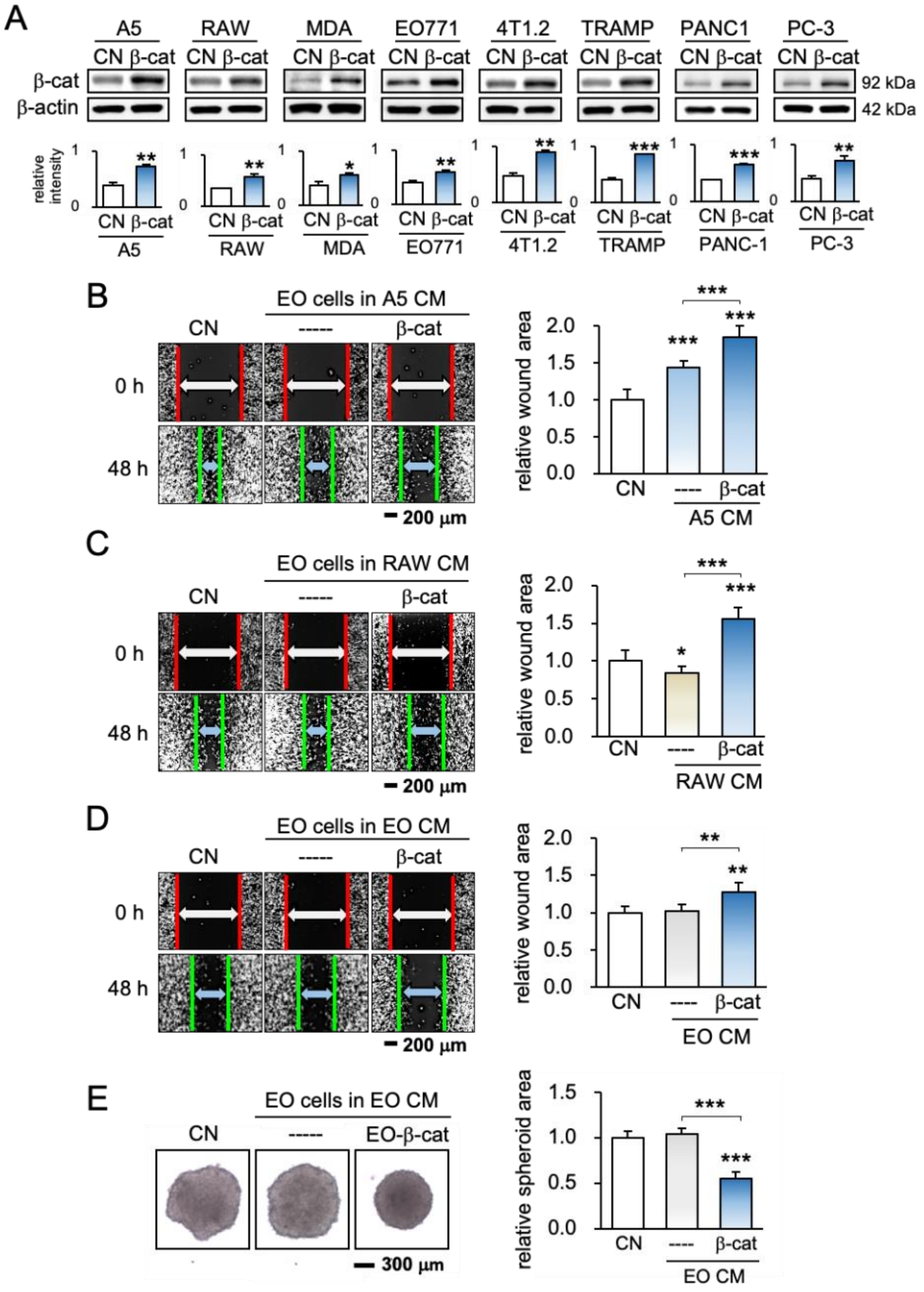

Suppl. Figure 1. Inhibition of migration and invasion by iTS cells. CM = conditioned medium, pCN = control plasmids, CN = control (no CM treatment), pβcat = β-catenin plasmid, A5 = MLO-A5 osteocytes, RAW = RAW 264.7 osteoclasts, and EO = EO771 mammary tumor cells. The double and triple asterisks indicate $p < 0.01$, and $p < 0.0001$, respectively. (A) Increased expression of β-catenin in tumor cells by expressing of β-catenin plasmid. (B-D) Inhibition of scratch-based migration of EO771 mammary tumor cells by β-catenin-overexpressing RAW264.7 osteocyte-derived, A5 osteocyte-derived, and EO771 tumor cell-derived iTS CM in 48 h, respectively. (E) Reduction in 3-dimensional spheroid growth of EO771 mammary tumor cells by EO771 cell-derived iTS CM in 72 h.

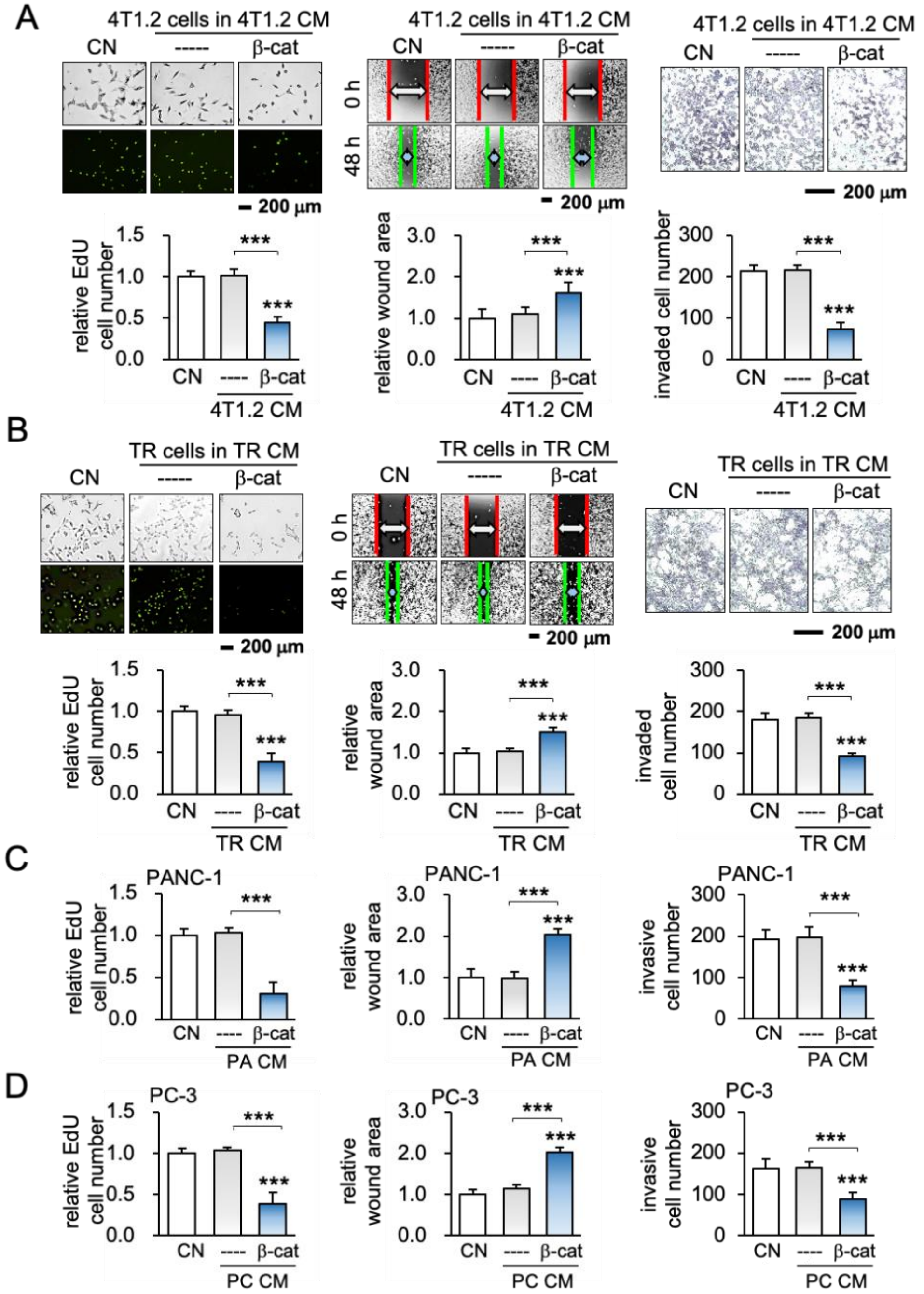

Suppl. Figure 2. Inhibition of proliferation, migration, and invasion by tumor cell-derived iTS CM. CM = conditioned medium, CN = control (no CM treatment), pβcat = β-catenin plasmids, and TR = TRAMP prostate tumor cells. The triple asterisk indicates $p < 0.0001$. (A) Inhibition of EdU-based proliferation, scratch-based migration, and Transwell invasion of 4T1.2 mammary tumor cells by β-catenin-overexpressing 4T1.2 tumor cell-derived iTS CM in 48 h. (B) Inhibition of EdU-based proliferation, scratch-based migration, and Transwell invasion of TRAMP prostate tumor cells by β-catenin-overexpressing TRAMP tumor cell-derived iTS CM in 48 h. (C&D) Inhibition of EdU-based proliferation, scratch-based migration, and Transwell invasion of PANC-1 pancreatic cancer cells, and PC-3 prostate cancer cells by their own iTS-CMs in 48 h.

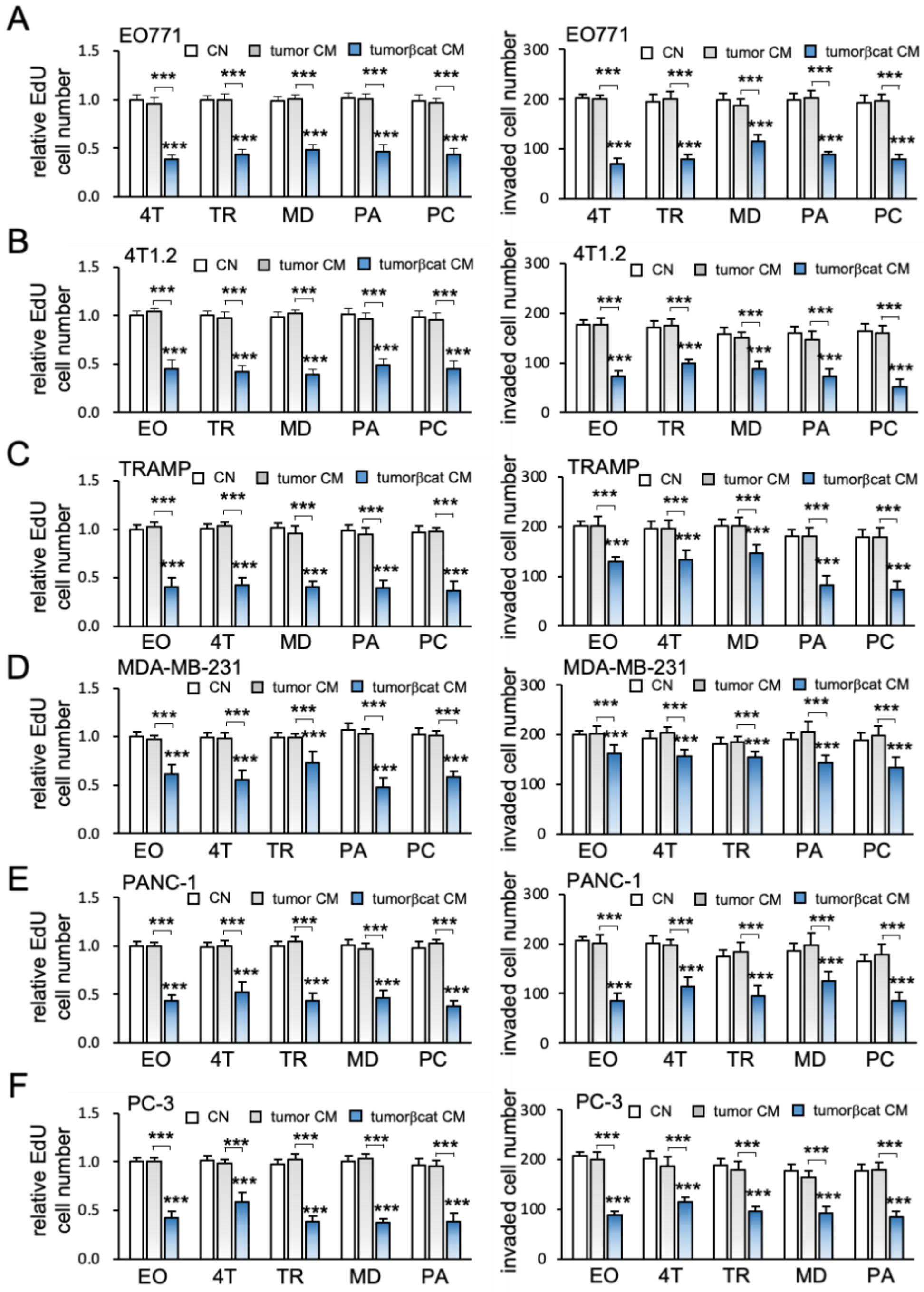

Suppl. Figure 3. Inhibition of proliferation and invasion by tumor cell-driven iTS CM. CM = conditioned medium, CN = control (no CM treatment), β-cat = β-catenin plasmids, EO = EO771 mammary tumor cells, 4T = 4T1.2 mammary tumor cells, TR = TRAMP prostate tumor cells. MD = MDA-MB-231 breast cancer cells, PA = PANC-1 pancreas cancer cells, PC = PC-3 prostate cancer cells, and the triple asterisk indicates $p < 0.0001$. Tumor cells to be inhibited include EO771 mammary tumor cells (A), 4T1.2 mammary tumor cells (B), TRAMP prostate cells(C), MDA-MB-231 breast cancer cells(D), PANC-1 pancreatic cancer cells (E), and PC-3 prostate cancer cells by β-catenin-overexpressing cohort cell-derived CM in 2 days.

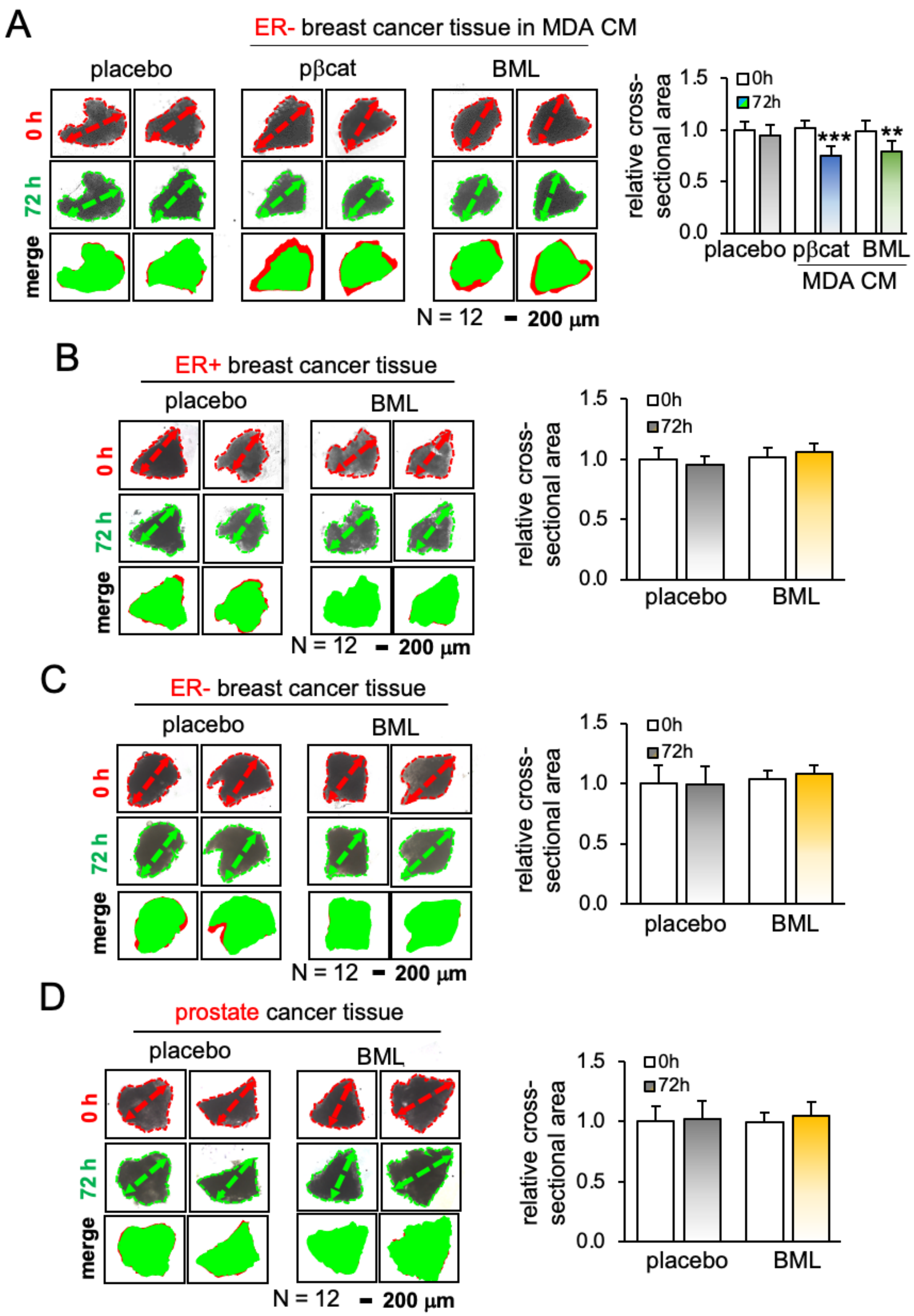

Suppl. Figure 4. Inhibition of ex vivo tumor growth by iTS CM with negative controls. CM = conditioned medium, pβcat = β-catenin plasmids, and MDA = MDA-MB-231 breast cancer cells. The double and triple asterisk indicates $p < 0.01$ and $p < 0.0001$, respectively. (A) Shrinkage of breast cancer tissue fragments (estrogen receptor-negative) by β-catenin-overexpressing, and BML284-treated MDA-derived iTS CM in 72 h. (B-D) Negative controls for BML284-treated tumor-derived iTS CM. No shrinkage of cancer fragments by direct treatment of three kinds of human cancer cells (estrogen receptor-positive and negative breast cancer tissues, and prostate cancer tissue, respectively) with BML284 in 72 h.

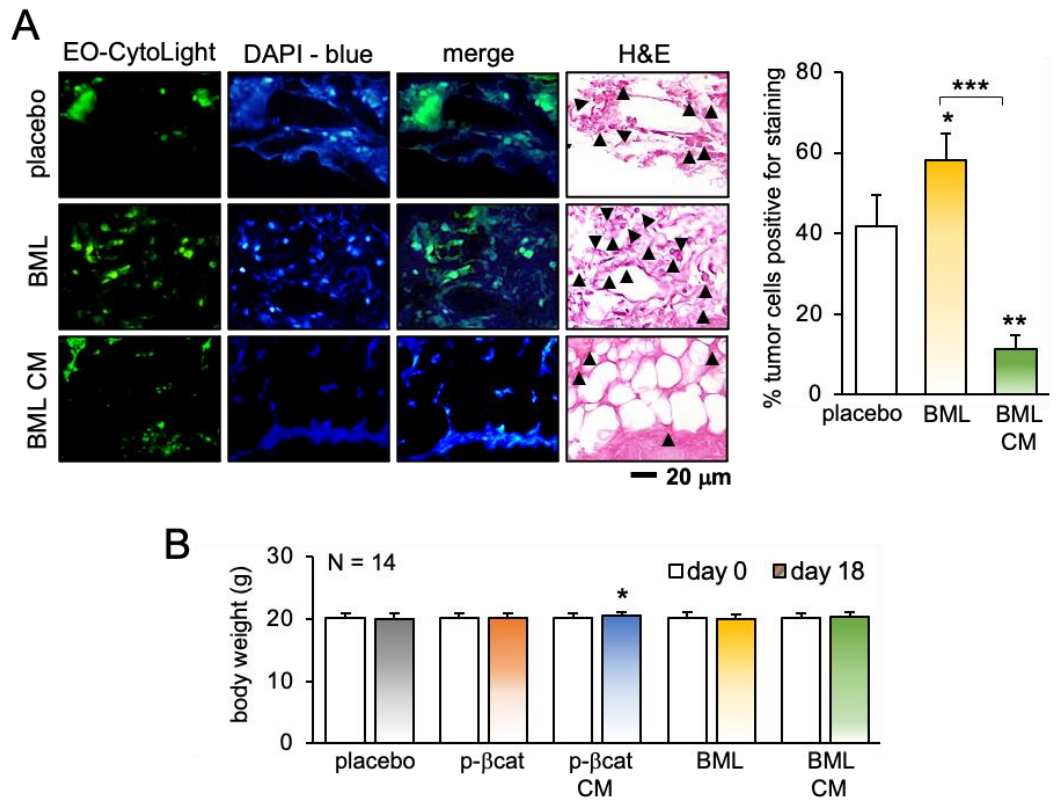

Suppl. Figure 5. Inhibition of tumor invasion by iTS CM in the mouse model with EO771 mammary tumor cells. CM = conditioned medium, and p βcat = β-catenin plasmids. The single, double and triple asterisks indicate $p < 0.05$, $p < 0.01$, and $p < 0.0001$, respectively. (A) Increase in the invaded cells by the systemic administration of BML284, and a decrease by the administration of BML-treated EO771 cell-derived CM. (B) Bodyweight on days 0 and 18 for mice with mammary tumors.

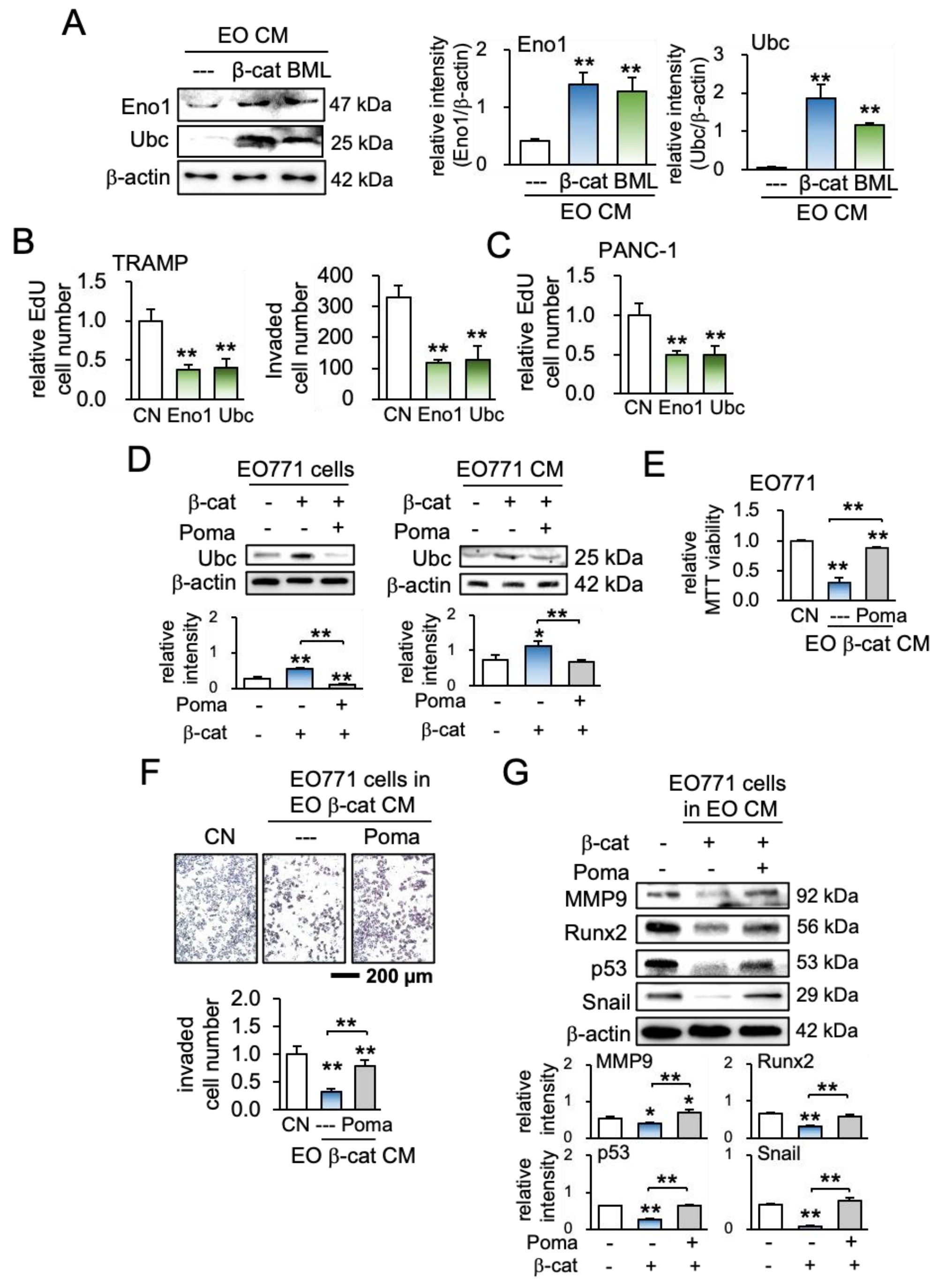

Suppl. Figure 6. Effect of enolase 1 and ubiquitin C on TRAMP prostate tumor cells and PANC-1 pancreatic tumor cells. CN = control, Eno1 = enolase 1, Ubc = ubiquitin C, CM = conditioned medium, β-cat = β-catenin plasmids, and Poma = Pomalidomide. The single, and double asterisks indicate $p < 0.05$, and $p < 0.01$, respectively. (A) Expression of enolase 1 and ubiquitin C in β-catenin-overexpressing and BML284-treated EO771 CMs. (B&C) Reduction in EdU-based proliferation and Transwell invasion of TRAMP prostate tumor cells and PANC-1 pancreatic tumor cells in response to enolase 1 and ubiquitin C. (D-G) Repressive effects of pomalidomide, an inhibitor of E3 ubiquitin ligase, on the reduced proliferation and invasion of EO771 cells by β-catenin overexpressing iTS CM.

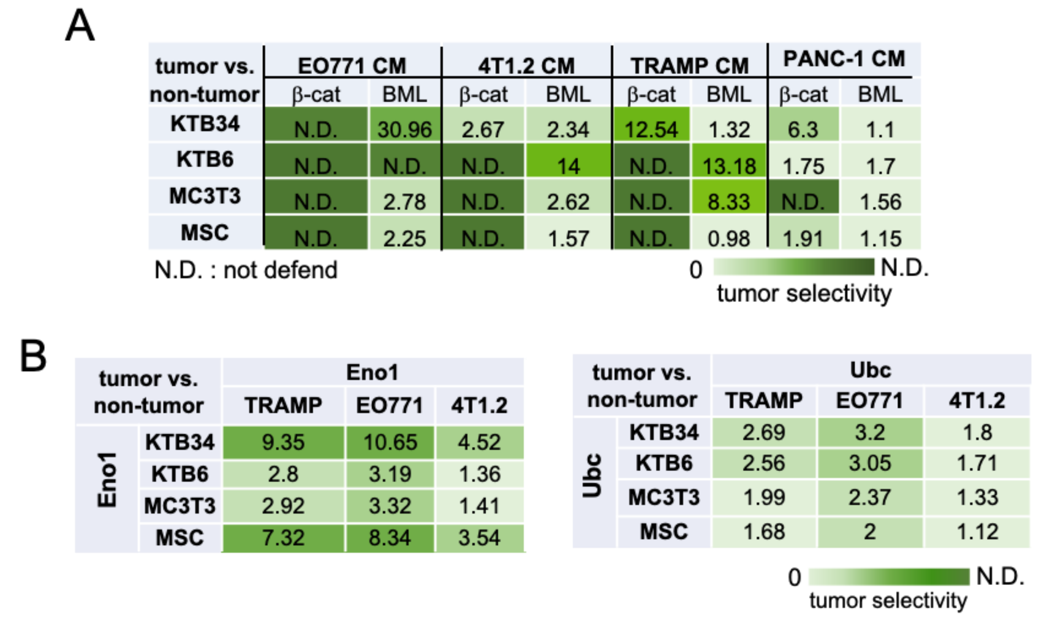

A

tumor vs. non-tumor	EO771 CM		4T1.2 CM		TRAMP CM		PANC-1 CM	
	β-cat	BML	β-cat	BML	β-cat	BML	β-cat	BML
KTB34	N.D.	30.96	2.67	2.34	12.54	1.32	6.3	1.1
KTB6	N.D.	N.D.	N.D.	14	N.D.	13.18	1.75	1.7
MC3T3	N.D.	2.78	N.D.	2.62	N.D.	8.33	N.D.	1.56
MSC	N.D.	2.25	N.D.	1.57	N.D.	0.98	1.91	1.15

N.D. : not defend

0 – N.D. tumor selectivity

B

tumor vs. non-tumor		Eno1 TRAMP	Eno1 EO771	Eno1 4T1.2
Eno1	KTB34	9.35	10.65	4.52
	KTB6	2.8	3.19	1.36
	MC3T3	2.92	3.32	1.41
	MSC	7.32	8.34	3.54

tumor vs. non-tumor		Ubc TRAMP	Ubc EO771	Ubc 4T1.2
Ubc	KTB34	2.69	3.2	1.8
	KTB6	2.56	3.05	1.71
	MC3T3	1.99	2.37	1.33
	MSC	1.68	2	1.12

0 – N.D. tumor selectivity

Suppl. Figure 7. Tumor selective inhibition was achieved by conditioned medium, enolase 1 and ubiquitin C. CM = conditioned medium, β-cat = β-catenin plasmids, Eno1 = Enolase 1, and Ubc = ubiquitin C. (A) In response to β-catenin-overexpressing iTS CM, tumor selectivity was above 1 or not defined (N.D. – the proliferation of non-tumor cells was stimulated. (B) Tumor selectivity of enolase 1 and ubiquitin C in 32 total cases, which present the comparisons among 4 tumor cell lines and 4 non-tumor cell lines.

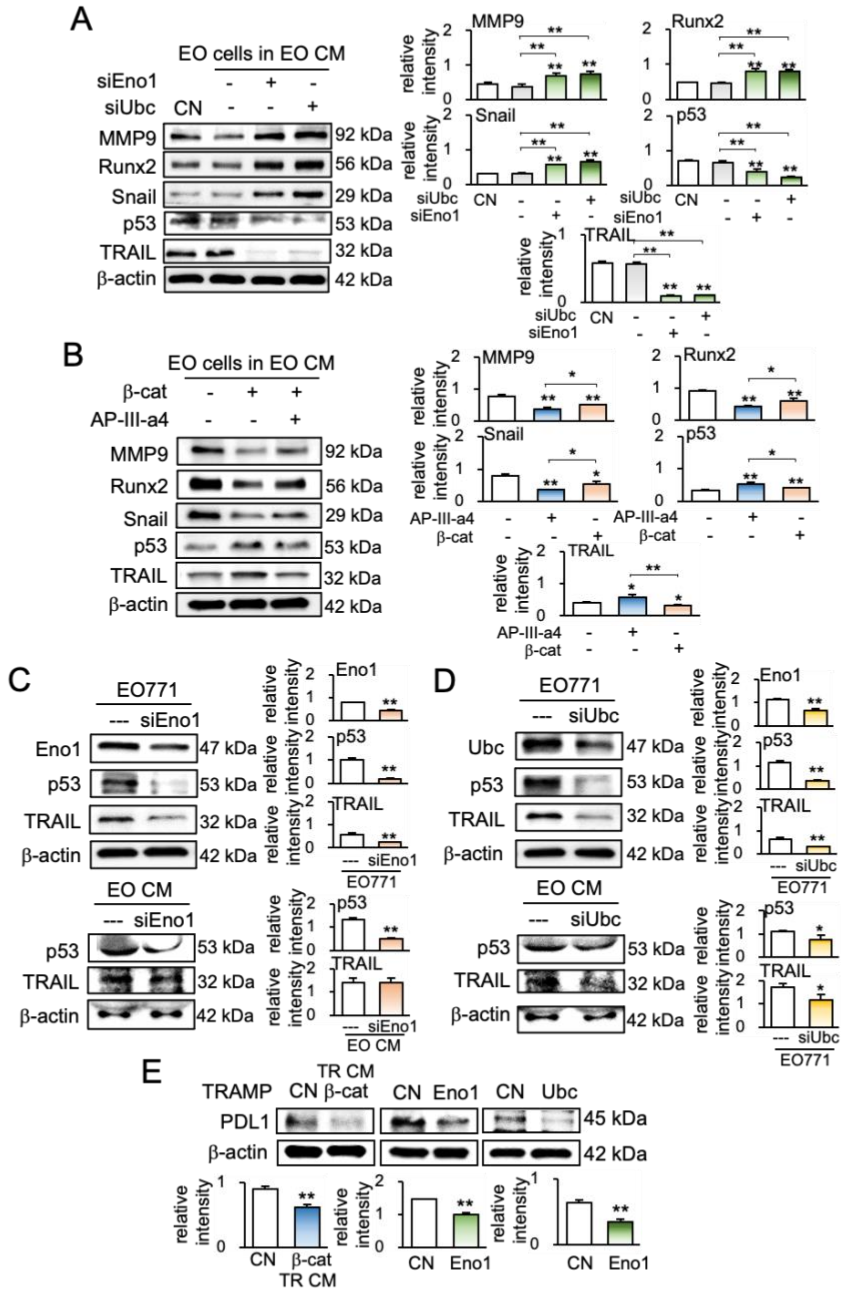

Suppl. Figure 8. Effects of enolase 1, ubiquitin C, and iTS CM on the expression of tumor-promoting and tumor-suppressing genes. CM = conditioned medium, CN = control (no CM treatment), β-cat = β-catenin plasmids, siEno1 = Enolase 1 siRNA, siUbc = ubiquitin C siRNA, EO = EO771 mammary tumor cells, and TR = TRAMP prostate tumor cells. (A) Expression of MMP9, Runx2, Snail, p53, and TRAIL in response to iTS CM after silencing enolase 1 and ubiquitin C. (B) Expression of MMP9, Runx2, Snail, p53, and TRAIL in response to EO771 cell-derived CM that was treated with an inhibitor of enolase 1 (AP-III-a4). (C&D) Decreased expression of p53 and TRAIL in EO771 cells and EO771-derived CM by silencing of enolase 1 and ubiquitin C. (E) Reduction of PDL1 in response to β-catenin- overexpressing iTS-derived CM, enolase 1, and ubiquitin C in TRAMP cells.

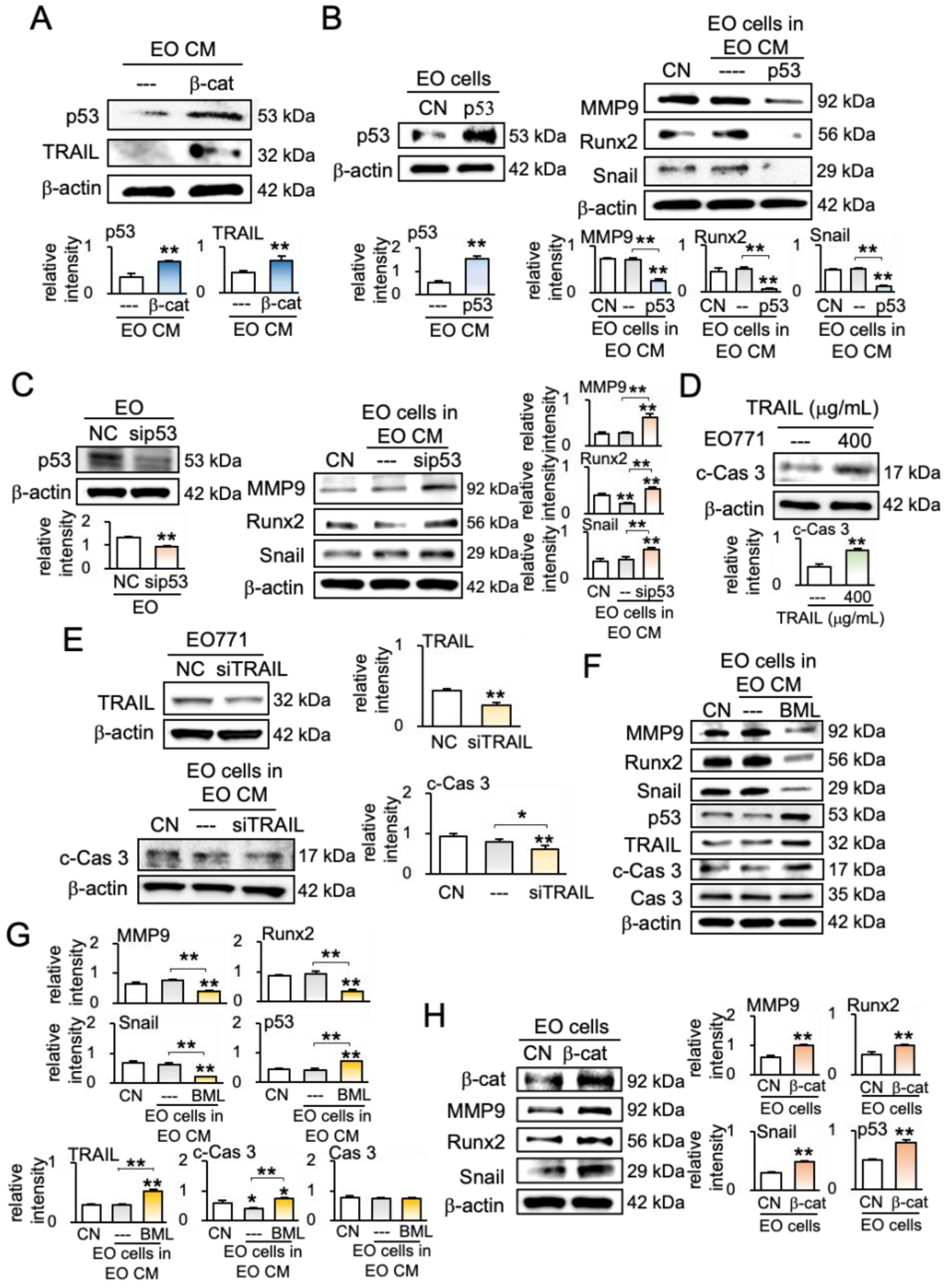

Suppl. Figure 9. Effects of p53 and TRAIL on regulatory mechanisms involved in iTS CM. CM = conditioned medium, CN = control (no CM treatment), si = siRNA, β-cat = β-catenin plasmids, and EO = EO771 mammary tumor cells. (A) Expression of p53, and TRAIL in β-catenin-overexpressing tumor cell-derived CM. (B) Expression of MMP9, Runx2, and Snail of EO771 mammary tumor cells in response to p53-overexpressing tumor cells-derived CM. (C) Upregulation of MMP9, Runx2, and Snail of EO771 mammary tumor cells in response to p53 silencing tumor cell-derived CM. (D) Expression of cleaved-caspase 3 in response to TRAIL in EO771 tumor cells. (E) Decreased expression of cleaved caspase 3 in EO771 cells by TRAIL silencing tumor cell-derived CM. (F&G) Expression of MMP9, Runx2, Snail, p53, TRAIL, and caspase 3 in EO771 mammary tumor cells in response to BML284 pre-treatment tumor cell-derived CM. (H) Expression of tumor-promoting genes in response to β-catenin in the EO771 mammary tumor cells.

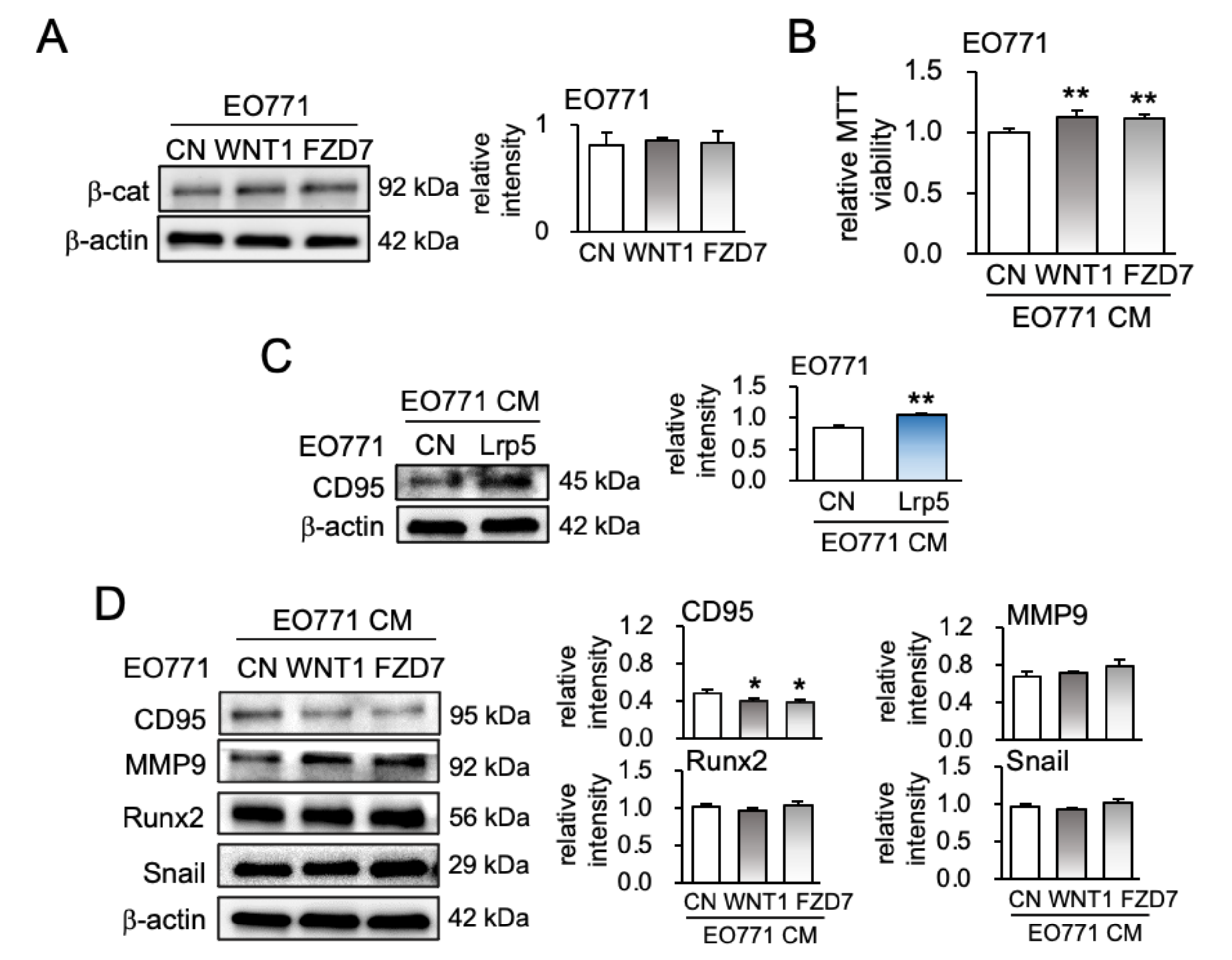

Suppl. Figure 10. Effects of WNT1- and FZD7-overexpressing EO771-derived CM on the proliferation of EO771 mammary tumor cells. CM = conditioned medium, CN = control, and β-cat = β-catenin. (A) No detectable change in the expression of b-catenin in EO771 cells in response to WNT1- and FZD7-overexpressing EO771-derived CM. (B) Elevation of MTT-based viability of EO771 tumor cells in response to WNT1- and FZD7-overexpressing EO771-derived CM. (C) Elevation of CD95 in EO771 cells in response to Lrp5-overexpressing EO771-derived CM. (D) Decreased level of CD95 and no detectable change in the expression of MMP9, Runx2, and Snail in EO771 cells in response to WNT1- and FZD7-overexpressing EO771-derived CM.

Suppl. Table 1. Pan-cancers gene in TCGA database

Cancer Type	No.of Samples	Explanation
ACC	79	Adrenocortical carcinoma
BLCA	408	Bladder Urothelial Carcinoma
BRCA	1095	Breast invasive carcinoma
CESC	304	Cervical squamous cell carcinoma and endocervical adenocarcinoma
CHOL	36	Cholangio carcinoma
COAD	449	Colon adenocarcinoma
DLBC	48	Lymphoid Neoplasm Diffuse Large B-cell Lymphoma
ESCA	184	Esophageal carcinoma
GBM	153	Glioblastoma multiforme
HNSC	520	Head and Neck squamous cell carcinoma
KICH	66	Kidney Chromophobe
KIRC	533	Kidney renal clear cell carcinoma
KIRP	290	Kidney renal papillary cell
LGG	515	Brain Lower Grade Glioma
LIHC	371	Liver hepatocellular carcinoma
LUAD	515	Lung adenocarcinoma
LUSC	501	Lung squamous cell carcinoma
MESO	87	Mesothelioma
OV	304	Ovarian serous cystadenocarcinoma
PAAD	178	Pancreatic adenocarcinoma
PCPG	179	Pheochromocytoma and Paraganglioma
PRAD	497	Prostate adenocarcinoma
READ	159	Rectum adenocarcinoma
SARC	259	Sarcoma
SKCM	103	Skin Cutaneous Melanoma
STAD	415	Stomach adenocarcinoma
TGCT	134	Testicular Germ Cell Tumors
THCA	505	Thyroid carcinoma
THYM	120	Thymoma
UCEC	532	Uterine Corpus Endometrial Carcinoma
UCS	57	Uterine Carcinosarcoma
UVM	80	Uveal Melanoma
